# Melatonin serves as a novel treatment in bladder fibrosis through TGF-β1/Smad and EMT

**DOI:** 10.1371/journal.pone.0295104

**Published:** 2024-03-13

**Authors:** Yang Zhang, Sun Gong, Weixin He, Jie Yuan, Di Dong, Jialong Zhang, Haomin Wang, Binghai Chen

**Affiliations:** 1 Department of Urology, Affiliated Hospital of Jiangsu University, Zhenjiang, Jiangsu, China; 2 Institute of Translational Medicine, Jiangsu University, Zhenjiang, Jiangsu, China; National Institutes of Health, UNITED STATES

## Abstract

**Background:**

Melatonin (MEL) is an indole amine molecule primarily produced in the pineal gland. Melatonin has been shown in numerous studies to have antifibrotic effects on the kidney, liver, and other organs. However, it is still unclear how melatonin works in bladder fibrosis. We explored how melatonin affects animals with bladder fibrosis and the underlying mechanisms.

**Materials and methods:**

MEL was used to treat human bladder smooth muscle cells (HBdSMCs) after they were stimulated with transforming growth factor-β1 (TGF-β1) *in vitro*. Proteomic analysis and bioinformatic analysis of the altered expression of these proteins were subsequently performed on HBdSMCs from the different processing methods. To construct an *in vivo* bladder fibrosis model, we injected protamine sulfate (PS) and lipopolysaccharide (LPS) twice a week into the rat bladder for six weeks. After two weeks of PS/LPS treatment, the mice in the treatment group were treated with MEL (20 mg/kg/d) for 4 weeks. Finally, we detected the expression of fibrosis markers from different perspectives. The TGF-β1/Smad pathway and epithelial–mesenchymal transition (EMT) in cell and bladder tissues were also identified. Further proteomic analysis was also performed.

**Results:**

*In vitro*, we found that TGF-β1 treatment enhanced the expression of the fibrosis markers collagen III and α-SMA in HBdSMCs. E-cadherin expression decreased while the TGF-β1/Smad pathway was activated. Vimentin and N-cadherin expression was also elevated at the same time. Similar findings were observed in the LPS group. After MEL treatment, the expression of collagen III and α-SMA decreased, the expression of E-cadherin increased, and the expression of vimentin and N-cadherin also decreased. According to our quantitative proteomics analysis, CCN1 and SQLE may be important proteins involved in the development of bladder fibrosis. MEL decreased the expression of these genes, leading to the relief of bladder fibrosis. Bioinformatics analysis revealed that the extracellular space structure related to metabolic pathways, actin filament binding, and stress fibers can serve as a pivotal focus in the management of fibrosis.

**Conclusion:**

Melatonin attenuates bladder fibrosis by blocking the TGF-β1/Smad pathway and EMT. CCN1 appears to be a possible therapeutic target for bladder fibrosis.

## 1. Introduction

Fibrosis is a progressive disease that features tissue scarring and is a typical and inevitable pathological outcome of many long-lasting inflammatory conditions. Fibrosis is closely associated with bladder dysfunction, including neurogenic bladder and bladder outlet obstruction (BOO) [1, 2]. In BOO patients, increased pressure in the bladder causes bladder detrusor hypertrophy. Bladder morphology may change irreversibly in untreated BOO patients, leading to increased collagen accumulation and bladder fibrosis [3].

The pleiotropic cytokine transforming growth factor (TGF) regulates a diverse array of biological processes, including the development of the extracellular matrix (ECM), differentiation of cells, and immunological control [4]. Transforming growth factor-β (TGF-β), the major fibrosis factor produced mostly by macrophages, emerges in the early stages of the wound healing response and has both anti-inflammatory and pro-fibrotic properties. In the liver, lung, kidney, skin, and heart, the development of fibrosis is correlated with the synthesis of TGF-β. The development of fibrosis has been demonstrated to be inhibited by suppression of the transforming growth factor-β1 (TGF-β1) signaling pathway in numerous animal models [5], and the TGF-β/Smad signaling pathway has drawn increased amounts of attention as a potent target for antifibrosis therapy [6].

The epithelial–mesenchymal transition (EMT) is regulated by epithelial–mesenchymal transition-regulating transcription factors (EMT-TFs), which suppress epithelial genes while activating the expression of mesenchymal components. Extracellular signals control EMT-TF expression, which frequently involves collaboration between several signaling pathways that modify epithelial plasticity in an environment-dependent manner. While a variety of signals can regulate EMT, TGF-β often plays a dominant role [7–9]. The EMT is recognized as a pivotal cellular mechanism contributing to the progression of tumors, fibrosis, and wound healing. Epithelial cells abandon their adhesion while adopting mesenchymal traits, including anteroposterior polarity, migratory capabilities, and the ability to invade adjacent tissues [10]. Basement membranes, other extracellular matrix (ECM) structures, and interactions with nearby cells are all altered by these alterations.

Melatonin (MEL) is a small indoleamine molecule. It is primarily synthesized by the pineal gland during physiologically normal conditions when the hypothalamus suprachiasmatic nucleus is activated at night [11]. Many studies have indicated that melatonin has antifibrotic effects on the liver, lungs, and various other organs [12]. However, the mechanism of the antifibrotic effect of melatonin has remained elusive. To date, there have been no reports on the function of melatonin in bladder fibrosis. Hence, our investigation focused on elucidating the inhibitory impact of melatonin on rat bladder fibrosis as well as its molecular mechanisms.

To validate our hypothesis, a series of experiments were conducted both *in vivo* and *in vitro*. Human bladder smooth muscle cells (HBdSMCs) with a fibrotic phenotype stimulated by TGF-β1 were treated with different concentrations of melatonin. To understand the role of melatonin in bladder fibrosis, the TGF-β1/Smad pathway and EMT, we used various methods to identify and evaluate the underlying mechanism *in vivo* and *in vitro*.

## 2. Materials and methods

### 2.1 Materials

Dimethyl sulfoxide (DMSO) was acquired from Solarbio Technology (Beijing, China). MEL (purity >95%, molecular weight: 232.28) and protamine sulfate (PS) were purchased from Sangon Bioengineering (Shanghai, China). TGF-β1 was acquired from Ucallm (Wuxi, China). Lipopolysaccharide (LPS) was acquired from Beyotime Biotechnology (ST1470; reagent grade) and purified by phenol extraction (Shanghai, China). Dulbecco’s modified Eagle medium (DMEM), phosphate-buffered saline (PBS), and forward-based medium (FBS) were acquired from XP Biomed (Shanghai, China). HBdSMCs were cultured at 37°C in 5% CO2 and 95% air. The antibodies used included phospho-Smad2 (cat. AF3362, Affinity), phospho-Smad3 (cat. AF3449, Affinity), Smad2/3 (cat. AF6367, Affinity), E-cadherin (cat. 13116, CST), vimentin (cat. 60330, Proteintech), collagen type III (cat. 22734, Proteintech), SQLE (cat. 12544, Proteintech), N-cadherin (cat. 3195, CST), α-smooth muscle actin (cat. 14395, CST), TGF-β1 (cat. 21898, Proteintech), and CCN1 (cat. AF3362, Affinity).

### 2.2 Animals

**Fig 1:**
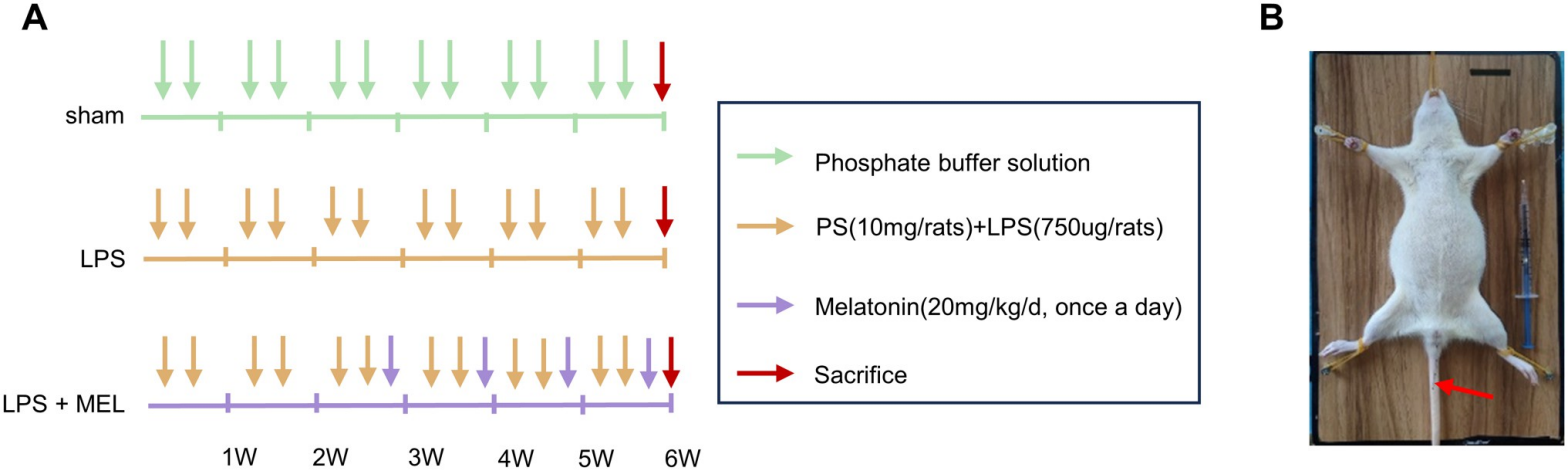
Establishment of rat bladder fibrosis model. (A) Schematic diagram of the experimental design for evaluating rat bladder fibrosis. (B) SD rat model (1 mm epidural catheter at the red arrow).

The Jiangsu University’s Ethics Committee and Animal Experiment Committee gave their permission for the study’s protocol. Eighteen 8-week-old female SD rats weighing approximately 200 grams were used in the experiment. Prior to the experiments, all the rats were acclimatized for one week in an environment with a temperature control of 21°C and a light-dark cycle of 12:12 hours. Animals had free access to food and water. The method described by Chae-Min Ryu et al. [13] was used to create a rat fibrosis model. All rats were randomly assigned to one of three groups (Fig 1A). For bladder emptying and drug administration, rats were catheterized using a 1 mm epidural catheter after isoflurane anesthesia (Fig 1B). The sham-operated group was perfused with 500 μL of PBS (sham group, n = 6); the PS/LPS treatment group was first perfused with PS (10 mg/rat, dissolved in 500 μL of PBS) to remove the epithelium, and the bladder was drained of PS by squeezing it after 45 min, followed by rinsing the bladder with PBS. Finally, LPS (750 μg/rat, dissolved in 500 μL of PBS) was instilled into the bladder through a catheter using a syringe, held for 30 min, drained by bladder pressure and rinsed with PBS after half an hour, twice a week (LPS group, n = 6). The same PS/LPS infusion method was used for the treatment group, but after two weeks of PS/LPS infusion, a specific dose of melatonin (20 mg/kg/day dissolved in 1% ethanol) was injected intraperitoneally every day for 4 weeks (LPS + MEL group, n = 6). At the end of the experiment, all rats were euthanized by isoflurane, and bladder tissue was removed for subsequent examination. Care was taken to maintain the body temperature of the rats during the experiment, and every effort was made to minimize pain.

### 2.3 Cell culture and treatments

HBdSMCs were procured from Otwo Biotech, Inc. (Guangzhou, China). To analyze the impact of melatonin on TGF-β1-induced HBdSMCs. First, we exposed the cells to TGF-β1 at different concentrations (5, 10, and 20 ng/mL) for 48 and 72 hours. After determining the optimal concentration (10 ng/mL) and duration (72 h) of treatment with TGF-β1, we investigated the therapeutic impact of melatonin on the fibrotic phenotype of the HBdSMCs. The cells were cotreated for 72 hours with various concentrations of melatonin (1, 10, 20, or 50 μM) and TGF-β1 (10 ng/mL). Finally, we determined the optimal TGF-β1 concentration (10 ng/mL) and melatonin concentration (10 ng/mL) and used these experimental conditions as the standards for subsequent experiments.

### 2.4 Cell viability analysis

In our *in vitro* experiments, melatonin was solubilized in DMSO. To assess the potential cytotoxicity of melatonin to human bladder smooth muscle cells, we treated the cells with varying concentrations of melatonin—1 μM, 10 μM, 20 μM, or 50 μM. Cell Counting Kit-8 (CCK8) (Vazyme, Nanjing, China) was used to assess TGF-β1 expression in cells 72 hours poststimulation.

### 2.5 Western blot analysis

HBdSMCs and rat bladder tissues were lysed with cell lysate (Beyotime, Jiangsu, China). Subsequently, the protein concentration was assessed and quantified using a bicinchoninic acid (BCA) assay (TaKaRa, Japan). Immunoblotting was conducted on cell and tissue extracts (30 μg total protein/well) via 10% sodium dodecyl sulfate‒polyacrylamide gel electrophoresis (SDS‒PAGE) (EpiZyme; PG113) and specific antibodies to determine the relative protein expression.

### 2.6 Reverse transcription-quantitative polymerase chain reaction (RT‒qPCR) assay

Total RNA was extracted from HBdSMCs treated with TGF-β1 and melatonin using a rapid RNA purification kit (Shanghai Yishan Biotech, Shanghai, China) according to the product instructions. Microultraviolet spectrophotometry was subsequently used to determine the RNA concentration and purity. Following quantification, HiScript III RT SuperMix was used to create cDNA from total RNA. For qPCR, a General-Purpose High Sensitivity Dye-based Quantitative PCR Assay Kit (Q711, Vazyme) was used to measure the appropriate concentrations of TGF-β1 and melatonin and to evaluate cell viability. With *GAPDH* as an internal reference, the relative expression level of the target gene was calculated using the 2^-△△Ct^ method. The sequences of primers used were as follows: *GAPDH* F: 5′- CGACCACTTTGTCAAGCTCA-3′, R: 5′-AGGGGTCTACATGGCAACTG-3′; target gene *Collagen III*: 5′- GATCAGGCCAGTGGAAATGT-3′, R: 5′- GTGTGTTTCGTGCAACCATC-3′; and target gene *α-SMA*: 5′- TTCAATGTCCCAGCCATGTA-3′, R: 5′- GAAGGAATAGCCACGCTCAG-3′.

### 2.7 Histological analysis

Bladder tissue was fixed in 10% formalin, embedded in paraffin wax and subsequently cut into continuous 5 μm thick sections. To evaluate rat bladder fibrosis, sections were stained using hematoxylin-eosin staining (HE) and Masson’s trichrome staining following standard protocols after dewaxing and washing. The sections were incubated with primary antibody at 4°C for immunohistochemical (IHC) staining, followed by washing, incubation with the secondary antibody coupled with HRP, and a final incubation at room temperature for an additional hour. Phospho-Smad2 (dilution 1:50, cat. AF3362, Affinity), phospho-Smad3 (dilution 1:50, cat. AF3449, Affinity), N-cadherin (dilution 1:50, cat. 13116, CST), vimentin (dilution 1:2500, cat. 60330, Proteintech), collagen type III (dilution 1:500, cat. 22734, Proteintech), E-cadherin (dilution 1:400, cat. 3195, CST), α-smooth muscle actin (dilution 1:1500, cat. 14395, Proteintech), TGF-β1 (dilution 1:200, cat. 21898, Proteintech), and CCN1 (dilution 1:100, cat. AF3362, Affinity) were used.

### 2.8 Statistical analysis

The results are presented as the mean ± SEM and were analyzed with GraphPad Prism 9.0. Intergroup differences were evaluated through the unpaired t test, one-way analysis of variance, and post hoc pairwise comparisons using Tukey’s test. P values less than 0.05 were considered to indicate statistical significance.

## 3. Results

### 3.1 Melatonin inhibits the TGF-β1-induced fibrotic phenotype in HBdSMCs

**Fig 2:**
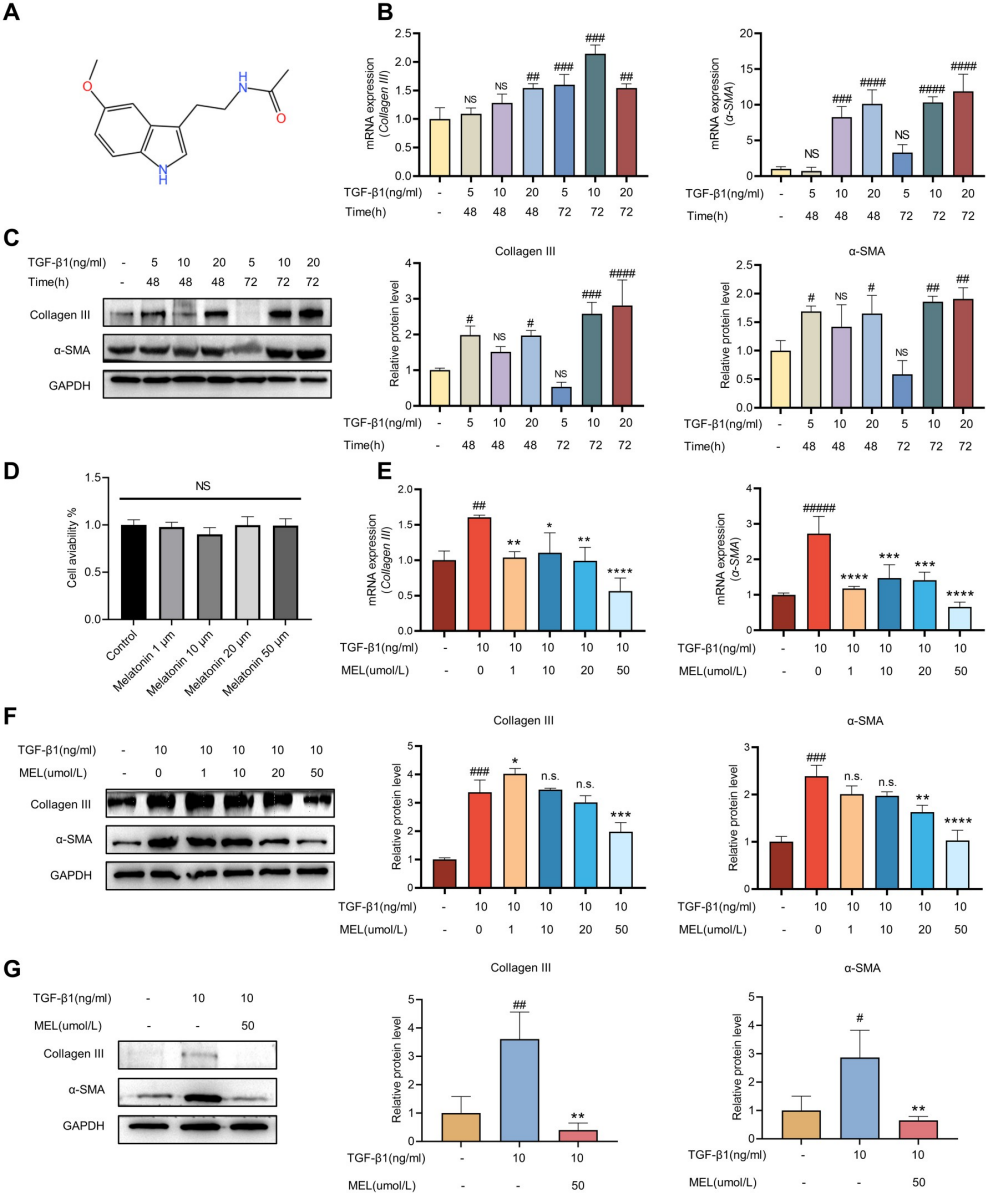
Effects of TGF-β1 on the expression of fibrosis indicators in HBdSMCs and the effects of MEL on the expression of fibrosis indicators in HBdSMCs induced by TGF-β1. (A) Chemical structural formula of melatonin. HBdSMCs were inoculated in six-well plates supplemented with different concentrations (5, 10 and 20 ng/mL) of TGF-β1 and incubated for 48 h or 72 h. The cells were processed immediately after they had reached the specified time points, and the protein type III collagen and α-SMA signal intensities relative to those of GAPDH were detected by RT‒qPCR (B) and western blot (C), respectively. (D) CCK-8 assay showing the effect of different concentrations of melatonin on the activity of HBdSMCs. In a previous experiment, we determined the optimal TGF-β1 concentration of 10 ng/mL and the optimal treatment time of 72 h. Then, HBdSMCs were inoculated into six-well plates within 10 generations, and different concentrations of melatonin were added (1 μM, 10 μM, 20 μM, 50 μM). Total RNA and protein were extracted after 72 h. The protein type III collagen and α-SMA signal intensities relative to those of GAPDH were detected by RT‒qPCR (E) and western blotting (F). (G)As described above, we selected a melatonin concentration of 50 μM as the optimal treatment concentration. For subsequent experiments, we used TGF-β1 (10 ng/mL, 72 h) and melatonin (50 μM) as the starting conditions, and on this basis, we detected the protein type III collagen and α-SMA signal intensities relative to those of GAPDH via western blotting. The data are shown as the mean ± SEM. #P<0.05, ##P<0.01, ###P<0.001, ####P<0.0001 vs. the control group. *P<0.05, **P<0.01, ***P<0.001, ****P<0.0001 vs. the TGF-β1 (10 ng/ml) group.

First, we prepare melatonin solutions of different concentrations according to the melatonin instructions (Fig 2A). To establish the TGF-β1-induced fibrosis model in HBdSMCs, cell culture was conducted with varying concentrations of TGF-β1 (5, 10, and 20 ng/mL) for different durations (48 h, 72 h). Hence, collagen III and α-SMA were used as reference markers for fibrosis, and their expression was evaluated via RT‒qPCR and immunoblotting analysis. The findings revealed that after treatment with 10 ng/mL TGF-β1 for 72 hours, the increase in collagen III was the most obvious. However, when the TGF-β1 concentration was increased to 20 ng/mL for 72 hours, the increase in the α-SMA level was most obvious (Fig 2B and 2C). First, fibrosis is dominated by increased collagen fibrin. Second, the normal range of urinary creatinine levels is approximately 20–275 mg/dL for women and 20–320 mg/dL for men. In an *in vivo* experiment in rats, we used inflammation to induce bladder fibrosis. To improve the accuracy of the overall experiment, we also simulated fibrosis caused by inflammatory stimulation in an *in vitro* model of bladder fibrosis. Studies have shown that the concentration of TGF-β1 in the urine of patients with inflamed urothelium (urocystitis) is 701.3 pg/mg creatinine [14]. In this context, when the TGF-β1 concentration is 10 ng/ml, the concentration is closer to the concentration of TGF-β1 in the urine of cystitis patients. Moreover, the relatively low stimulation concentration also prevents the unknown effects of excessive TGF-β1 stimulating factors on cells. Therefore, we used a TGF-β1 concentration of 10 ng/mL and a culture time of 72 h as the best reference conditions for subsequent fibrosis models. In the present study, melatonin was dissolved in DMSO, and certain concentrations of DMSO had toxic effects on the cells. To investigate whether melatonin is cytotoxic to human bladder smooth muscle cells, we treated human bladder smooth muscle cells with melatonin (1 μM, 10 μM, 20 μM, or 50 μM). The CCK-8 assay showed that melatonin had no significant cytotoxic effect on human bladder smooth muscle cells at the above concentrations (Fig 2D). After determining the optimal concentration (10 ng/mL) and duration (72 h) of treatment with TGF-β1, we investigated the therapeutic impact of melatonin on the fibrotic phenotype of the HBdSMCs. HBdSMCs induced by TGF-β1 (10 ng/mL, 72 h) were subjected to melatonin treatment at concentrations of 1, 10, 20, and 50 μM. RT‒qPCR and immunoblot analysis demonstrated that, after treatment with 50 μmol/L melatonin, the collagen III and α-SMA levels exhibited the most substantial decreases (Fig 2E and 2F). To corroborate the impact of melatonin on TGF-β1-induced fibrosis, we specifically examined the effect of melatonin at a dose of 50 μmol/L (Fig 2G).

### 3.2 Melatonin regulates the TGF-β1/Smad pathway and EMT

**Fig 3:**
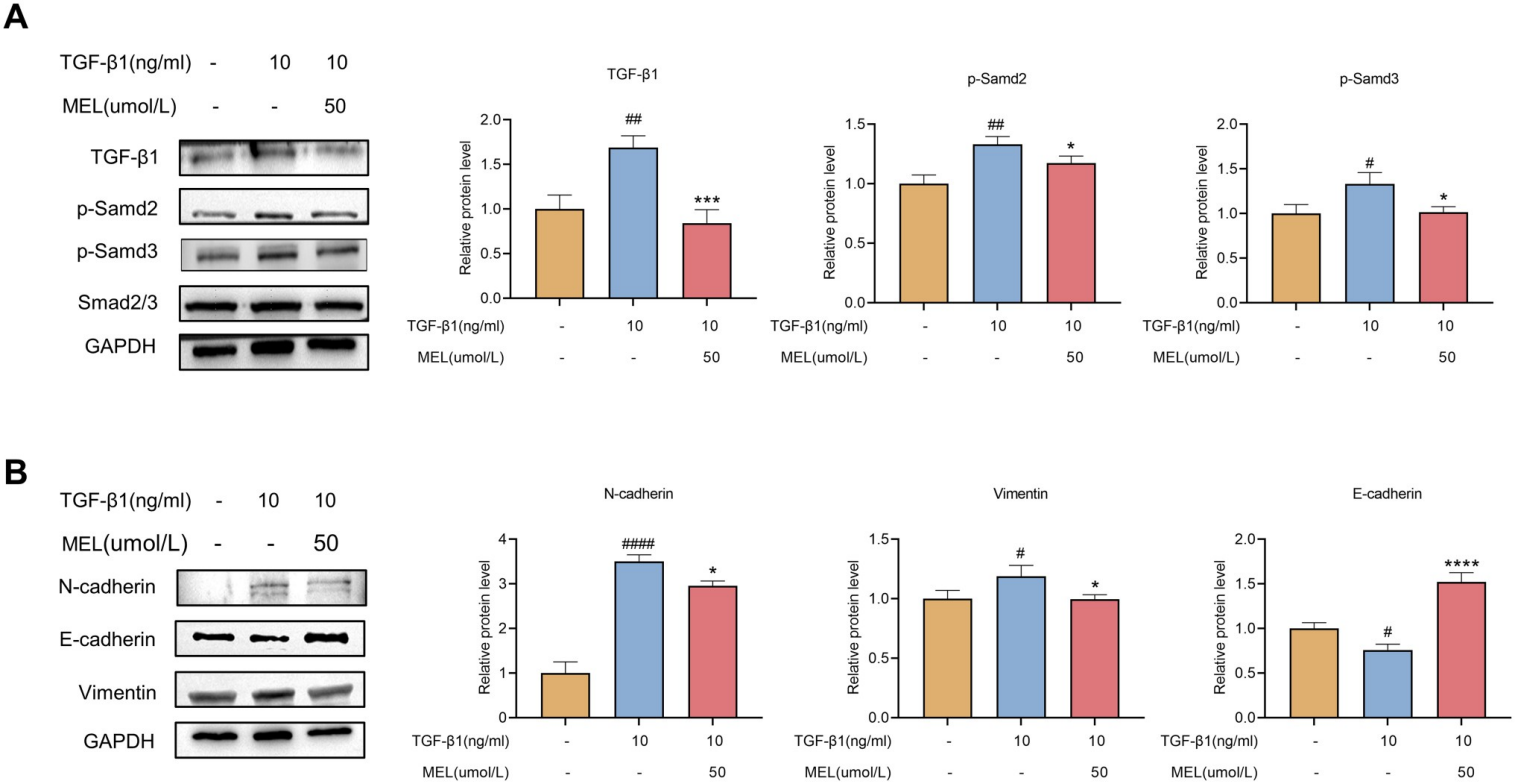
Effect of melatonin (50 μM) on the TGF-β1 (10 ng/mL, 72 h)-mediated TGF-β1/Smad pathway and EMT in HBdSMCs. HBdSMCs within 10 generations were inoculated in six-well plates, and the experiments were performed under the above conditions. Proteins were extracted after 72 h, and the expression levels of TGF-β1, p-Smad2, p-Smad3, and Smad2/3 (A) and of E-cadherin, N-cadherin, and Vimentin (B) were detected via western blotting. The data are shown as the mean ± SEM. #P<0.05, ##P<0.01, ###P<0.001, ####P<0.0001 vs. the control group. *P<0.05, **P<0.01, ***P<0.001, ****P<0.0001 vs. the TGF-β1 group.

To elucidate the mechanism by which melatonin inhibits TGF-β1-induced fibrosis in HBdSMCs, we conducted a comprehensive investigation into the alterations in the TGF-β1/Smad signaling pathway and EMT. The ideal TGF-β1 induction conditions (10 ng/mL, 72 hours) and melatonin treatment concentration (50 μmol/L) were used to validate subsequent research. Evaluations of the expression of the relevant proteins were performed. P-Smad2 and P-Smad3 expression was greater in the model group than in the untreated group, and melatonin (50 μmol/L) significantly decreased the expression of these proteins (Fig 3A). Smad2/3 expression did not change in response to any of the treatment factors. Compared to those in untreated cells, the expression of vimentin and N-cadherin in the model set increased, but that of E-cadherin decreased (Fig 3B). Melatonin (50 μmol/L) significantly reversed the changes in the expression of these proteins. According to our results, melatonin substantially reduces TGF-β1-induced fibrosis in HBdSMCs by modifying the TGF-β1/Smad pathway and EMT.

### 3.3 LPS induced bladder fibrosis in rats

**Fig 4:**
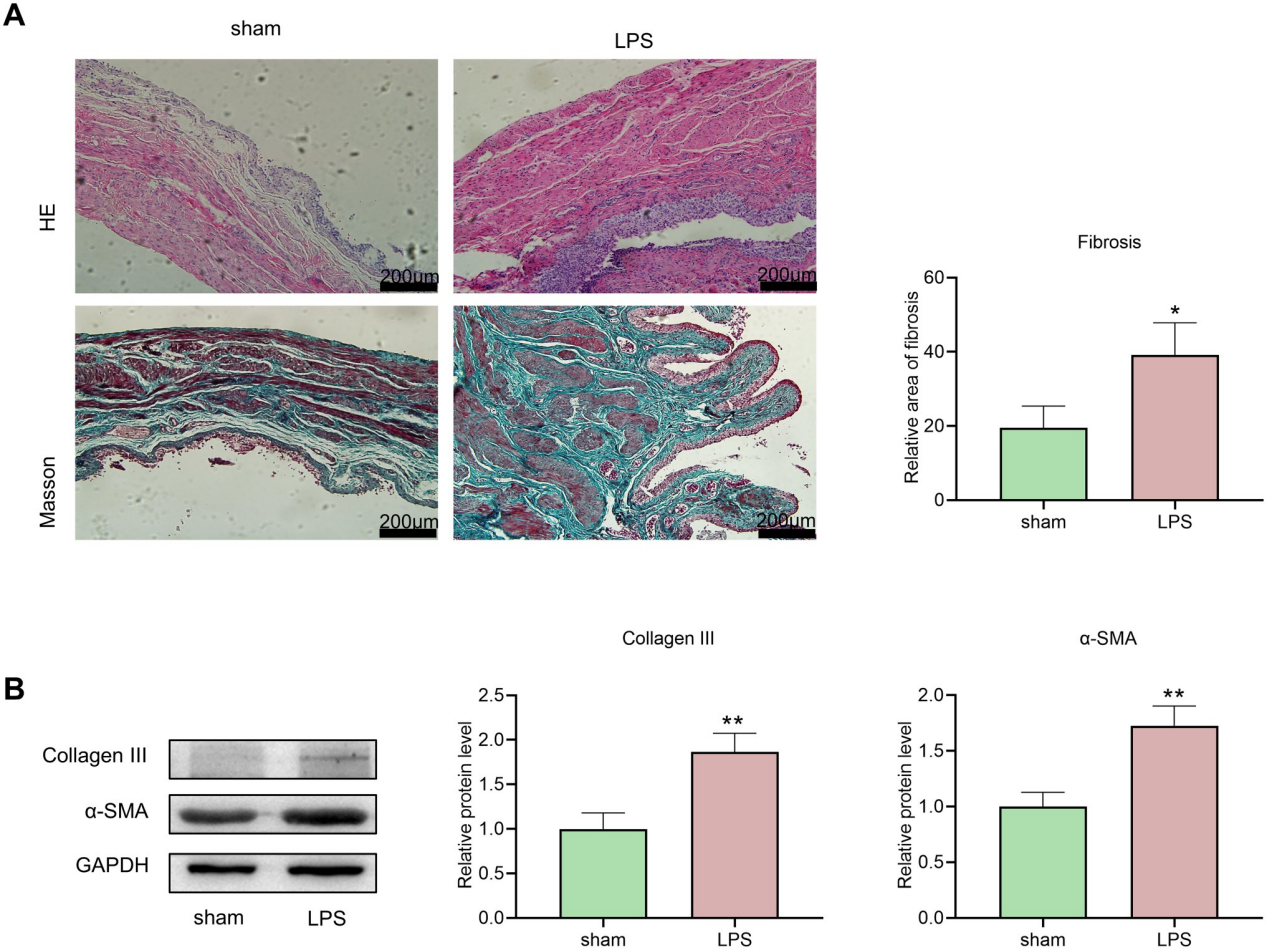
Histologic and protein changes in the PS/LPS-induced bladder fibrosis model in rats. (A) HE staining (obvious change in bladder thickness) and Masson trichrome staining (collagen fibers in blue) of bladder tissues from rats subjected to different treatments (100X magnification; scale bar, 200 μm). (B) Immunoblot analysis of the expression of collagen III and the α-SMA signal intensity relative to that of GAPDH in PS/LPS-induced rat bladder tissue. The data are shown as the mean ± SEM. *P<0.05, **P<0.01 vs. the sham group.

A dual infusion of PS and LPS was used to induce bladder tissue fibrosis in the rats. To assess whether this led to bladder wall hypertrophy and fibrogenesis, we conducted histological and morphometric analyses using HE and Masson trichrome staining. After 6 weeks of perfusion, HE staining revealed notable atypical hyperplasia of the bladder epithelium and hyperplasia of connective tissue in the bladder submucosa compared to those in the sham group. Masson’s trichrome staining indicated that the LPS group exhibited greater bladder smooth muscle hypertrophy and conspicuous collagen deposition than did the sham group (Fig 4A), which was consistent with the findings of Ryu and colleagues [13].

This outcome was corroborated through immunoblotting (Fig 4B). Thus, these data suggest that double instillation of LPS can effectively induce fibrosis in rat bladder tissue.

### 3.4 Melatonin reduces PS/LPS-induced bladder fibrosis in rats

**Fig 5:**
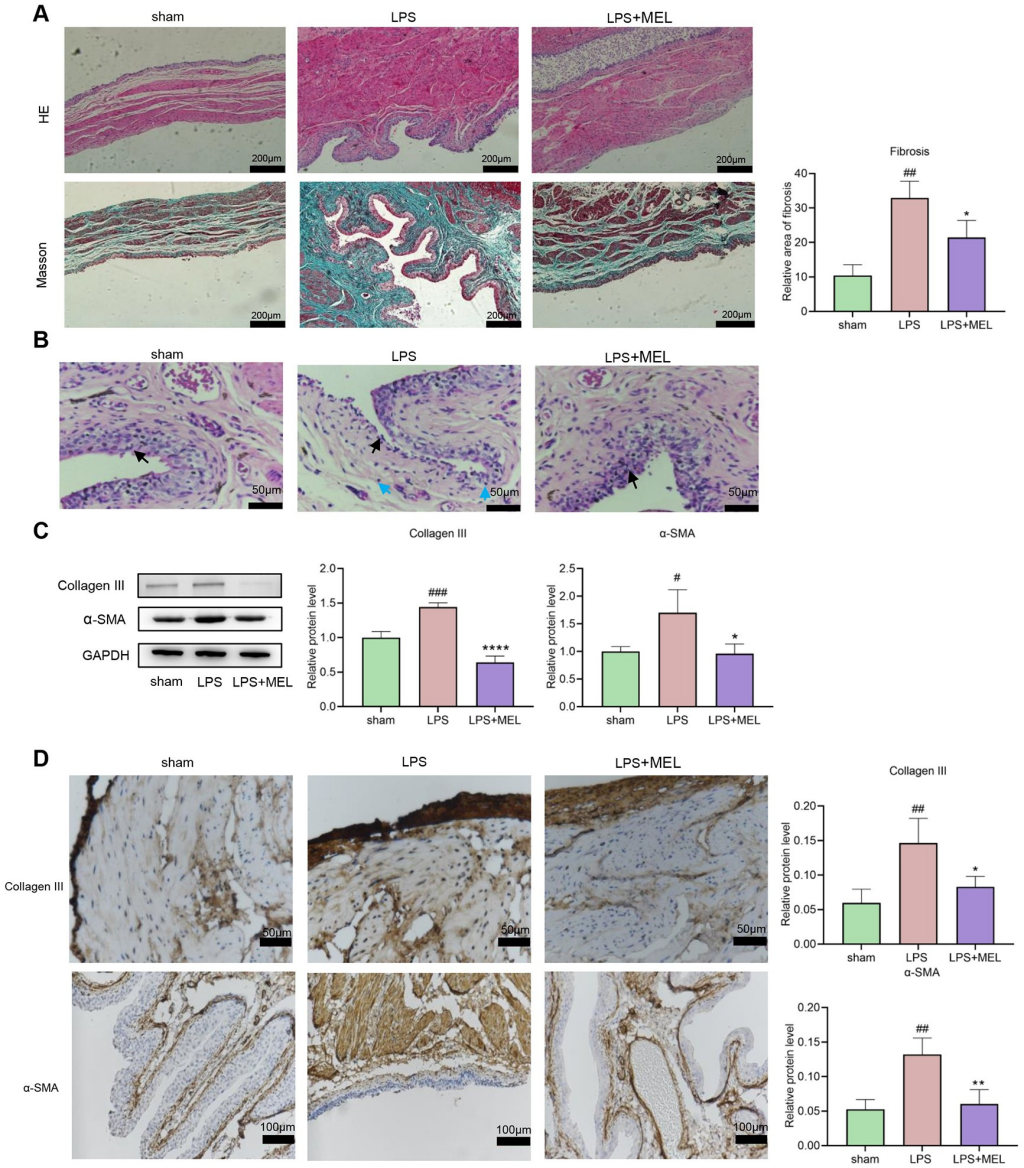
Therapeutic effect of melatonin on PS/LPS-induced bladder fibrosis in rats. (A) Rat bladder tissue under different experimental conditions. Representative H&E and Masson trichrome staining results. (100X magnification; scale bar, 200 μm) (B) In the sham group, the bladder epithelial cells are arranged in an orderly manner, with many layers and no obvious degeneration and shedding, as shown by the black arrow; in the LPS group, the bladder structure is abnormal, with some mucosal epithelial cells falling off, and the membrane *propria* is exposed, as shown by the black arrow; inflammation can be seen Cellular infiltration, as indicated by blue arrows. In the treatment group, the arrangement of bladder epithelial cells was slightly disordered, but no obvious degeneration and shedding was seen, as shown by the black arrow. (400X magnification; scale bar, 50 μm) (C) Western blot analysis of collagen III and α-SMA expression in rat bladder tissues under different experimental conditions. (D) Representative immunohistochemical results of collagen III and α-SMA in rat bladder tissues under different experimental conditions. (Collagen III, 400X magnification; Scale Bar, 50 μm. α-SMA, 200X magnification; Scale Bar, 100 μm). The data are shown as the mean ± SEM. #P<0.05, ##P<0.01, ###P<0.001 vs. the control group. *P<0.05, **P<0.01 vs. the LPS group.

We assessed the effect of melatonin on the histological changes induced by LPS double perfusion. First, in the current model, HE in the LPS instillation group showed marked atypical epithelial hyperplasia of the bladder and connective tissue hyperplasia of the bladder submucosa, which was consistent with our previous results. H&E and Masson staining following melatonin treatment revealed a significant reduction in atypical epithelial hyperplasia of the bladder and connective tissue hyperplasia in the bladder submucosa compared to those in the LPS group, suggesting that melatonin mitigated bladder fibrosis (Fig 5A and 5B). In the H&E-stained sections, under greater magnification, we observed that the degree of inflammation in the rat bladder model group increased significantly and then decreased significantly after melatonin treatment (Fig 5B). Immunoblotting and immunohistochemistry were used to measure the expression of associated proteins. Collagen III and α-SMA expression levels significantly decreased in the sham group following LPS/PS treatment. After melatonin administration, the relative protein expression was noticeably reduced (Fig 5C and 5D). These findings suggest that melatonin effectively mitigates LPS/PS-induced bladder fibrosis in rats.

### 3.5 Melatonin regulates the TGF-β1/Smad signaling pathway and EMT in PS/LPS-induced fibrotic bladders in rats

**Fig 6:**
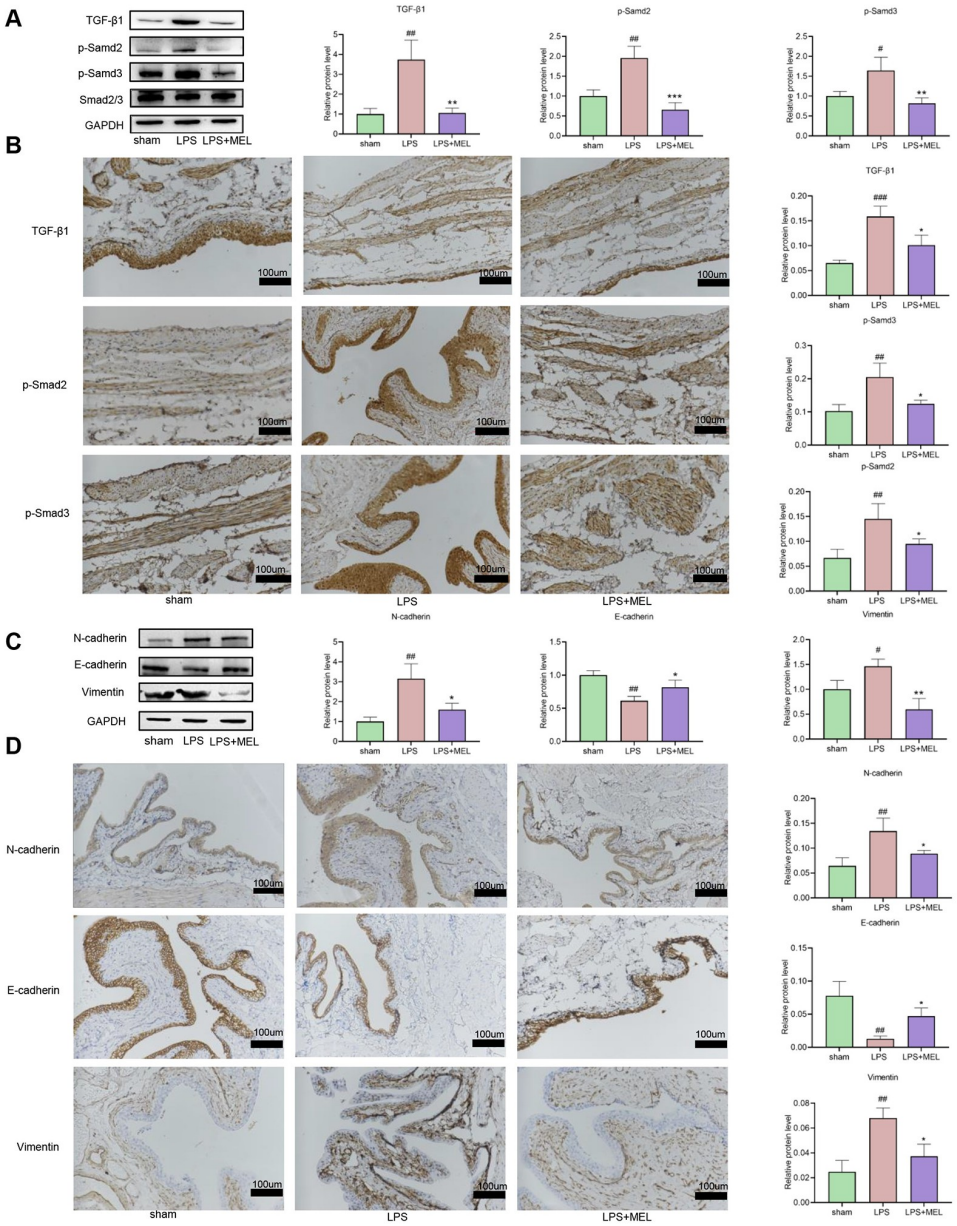
Effects of melatonin on the TGF-β1/Smad pathway and EMT in a PS/LPS-induced bladder fibrosis model in rats. (A, B) Representative western blot and IHC results for TGF-β1, p-Smad2, p-Smad3 and Smad2/3 in rat bladder tissues under different experimental conditions. (200X magnification; Scale Bar, 100 μm). (C, D) Representative western blot and IHC results for E-cadherin, N-cadherin and vimentin in rat bladder tissues under different experimental conditions. (200X magnification; scale bar, 100 μm). The data are shown as the mean ± SEM. #P<0.05, ##P<0.01 vs. the control group. *P<0.05, **P<0.01, and ***P<0.001 vs. the LPS group.

To further investigate the impact of melatonin on the TGF-β1/Smad signaling pathway and EMT in rat bladder tissues, we measured the expression of related proteins using immunohistochemistry and immunoblot analysis. TGF-β1, p-Smad2 and p-Smad3 expression was considerably increased after PS/LPS stimulation compared to that in the sham-operated group, whereas it was significantly lower after melatonin therapy (Fig 6A). Smad2/3 expression was constant across all groups. Similar findings were obtained from an immunohistochemical study (Fig 6B). These results indicate that the degree of fibrosis in the rat bladder fibrosis model can be attenuated by melatonin through decreasing the expression of the TGF-β1/Smad signaling pathway. To determine the status of EMT, we identified pertinent proteins in bladder tissues using immunohistochemical staining and immunoblot analysis. These findings demonstrated that whereas the expression of E-cadherin was lower in the LPS group than in the sham group, the levels of N-cadherin and vimentin were greater. Compared to those in the LPS group, the expression of these proteins was reversed in the bladder tissues of the melatonin-treated rats (Fig 6C and 6D). The above findings showed that melatonin can significantly slow EMT and ameliorate fibrosis in a PS/LPS fibrosis model, which highlights the importance of this mechanism.

### 3.6 Quantitative proteomic analysis of cells with an increased fibrosis phenotype and melatonin treatment

**Fig 7:**
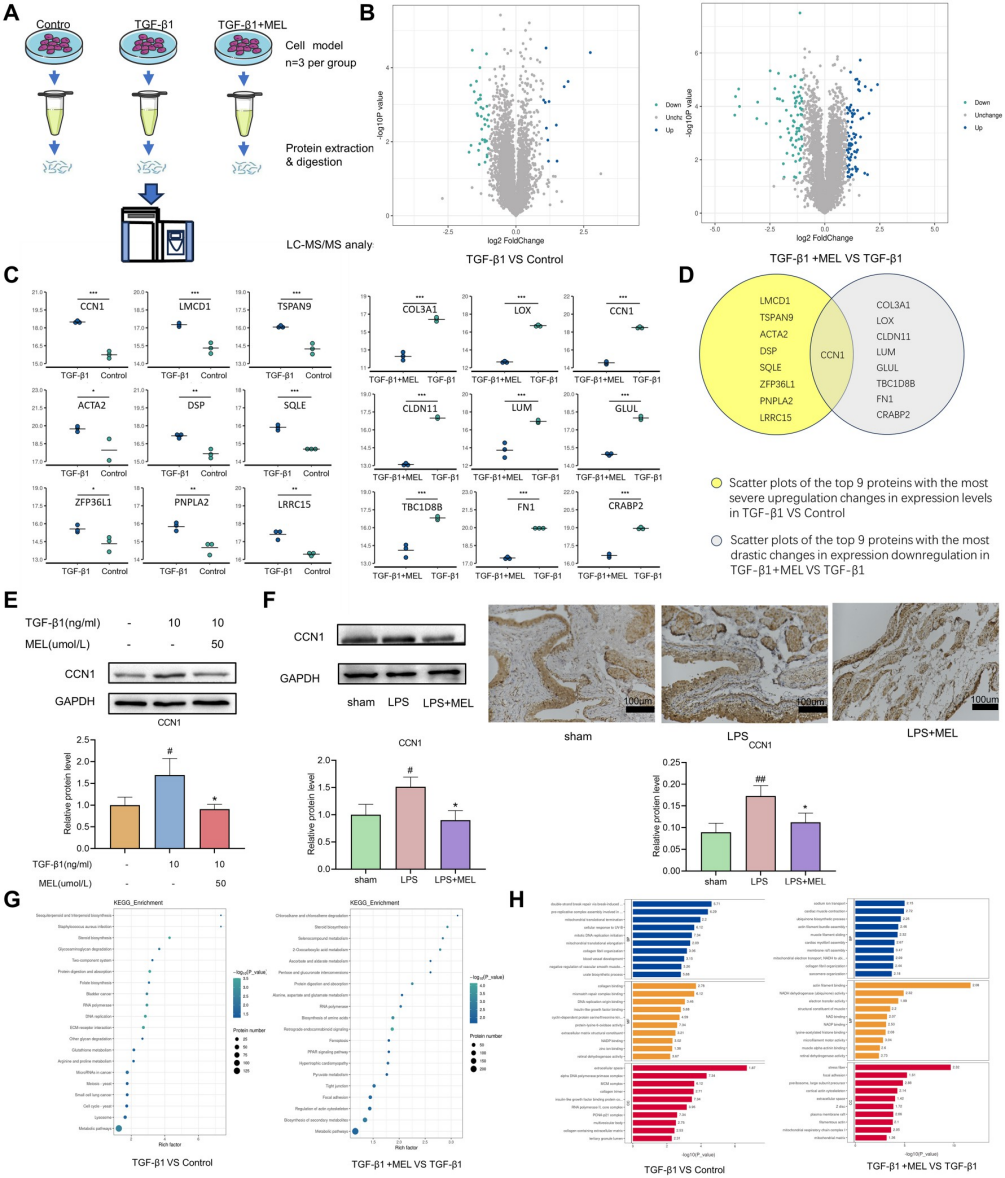
Proteomic analysis of control, TGF-β1 model, and melatonin-treated HBdSMCs. (A) Workflow diagram of the label-free quantitative proteomics experiments. Total proteins were extracted from different groups of cells (control group, TGF-β1 model group and melatonin group, n = 3 each) and digested with trypsin. Equal amounts of separated peptides were analyzed via LC‒MS/MS. A specific protein database was used for bioinformatics analysis of the differentially expressed proteins. (B) The results of differential protein screening are displayed in the form of a volcano plot. In the figure, blue denotes upregulated proteins, cyan denotes downregulated proteins, and gray‒black denotes no difference. The abscissa is the fold change (base 2 log transformation). The ordinate is the P value (base 10 log-transformed). (C) The top 9 most upregulated proteins between the TGF-β1 model and control groups and the top 9 most downregulated proteins between the MEL treatment group and the TGF-β1 model. (D) CCN1 may be a target protein for melatonin treatment. (E) Representative western blot showing CCN1 expression in HBdSMCs under different experimental conditions. (F) Representative western blot and IHC results of CCN1 in rat bladder tissues under different experimental conditions. (200X magnification; scale bar, 100 μm). (G) Bar graphs showing the results of GO enrichment analysis of the differentially expressed proteins. (H) The top 20 pathways enriched in the KEGG enrichment analysis results. The data are shown as the mean ± SEM. #P<0.05, ##P<0.01 vs. the control group. *P< 0.05, **P<0.01 vs. the model group.

To decipher the proteins and biological pathways implicated in the antifibrotic effects of melatonin, we conducted proteomic analysis using human bladder smooth muscle cells from the control group, TGF-β1 group, and TGF-β1+ MEL group (n = 3 for each group; Fig 6A). A total of 6100 nonredundant proteins were quantified via liquid chromatography-tandem mass spectrometry (LC‒MS/MS)-based proteomics experiments (S1 Table). All the groups exhibited good biological reproducibility, as evidenced by the Pearson correlation coefficient, which was used to measure the correlation between any two samples in each group and was greater than 0.94 (S1A Fig). A comparison of the TGF-β1 simulation group and the control group revealed substantial changes in 832 proteins. A total of 212 of these proteins were upregulated, whereas 620 were downregulated (Fig 7B). Between the MEL treatment group and the TGF-β1 model, 1564 proteins were substantially differentially expressed, of which 996 proteins were elevated and 568 proteins were downregulated. Subsequently, we identified the 9 proteins exhibiting the most substantial increase in expression in the TGF-β1 model. Similarly, we identified the 9 proteins related to the greatest decrease in expression in the MEL-treated group compared to the TGF-β1 model (Fig 7C). By combining these two sets of data (Fig 7D), we found that cellular communication network Factor 1 (CCN1) was the most likely target protein of melatonin treatment. Western blot analysis was used to evaluate CCN1 expression in the TGF-β1 model (Fig 7E). Remarkably, CCN1 expression was significantly heightened in the LPS groups (Fig 7F). In contrast, CCN1 expression was diminished in melatonin-treated human bladder smooth muscle cells and in rat bladder tissue. Immunohistochemical analysis corroborated the western blot findings, which revealed an increase in CCN1 expression in the LPS group, followed by a decrease postmelatonin treatment (Fig 7F). These findings support CCN1 as a pivotal protein in bladder fibrosis progression, and melatonin has been shown to be effective at reducing CCN1 expression and ameliorating fibrosis severity. Moreover, we also detected another interesting gene, squalene epoxidase (SQLE). Then, we performed western blot analysis of tissues and cells treated via different methods. We found that this protein was upregulated to varying degrees in tissues and cells with a tendency toward fibrosis. After MEL treatment, there were also varying degrees of decrease (S1B Fig).

Comprehensive enrichment analysis of Gene Ontology (GO) functional annotations revealed that the proteins exhibiting differential expression before and after TGF-β1 treatment were primarily associated with the extracellular space in terms of cellular composition. In contrast, the differentially expressed proteins before and after melatonin treatment were predominantly associated with actin filament binding and stress fiber-related molecular functions in the cellular composition (Fig 7G). Moreover, through Kyoto Encyclopedia of Genes and Genomes (KEGG) pathway enrichment analysis of the differentially expressed proteins among the various treatment groups, we observed that these proteins were primarily involved in metabolic pathways (Fig 7H). Hence, these results lead us to speculate that addressing the structural aspects of the extracellular space linked to metabolic pathways, actin filament binding, and stress fibers could be crucial focal points for fibrosis treatment.

## 4. Discussion

Bladder fibrosis is a common pathological process related to many urinary system diseases. In addition to benign prostatic hyperplasia-related BOO, many other diseases, such as aging stroke, Parkinson’s disease, multiple sclerosis, and diabetes, have been associated with bladder fibrosis [15]. Cystic fibrosis is a challenging issue that has not yet been solved since bladder function is weakened and difficult to recover from long-term chronic harm to the bladder [16]. The rat bladder fibrosis model in this study was generated via the PS/LPS double perfusion method established by Chae-Min Ryu et al. [13], and two weeks of perfusion was added on this basis because the histological appearance of this model was more consistent with that of Hunner type IC/BPS-related bladder lesions and more meaningful for clinical treatment.

Bladder fibrosis manifests as the proliferation of bladder smooth muscle cells (BSMCs) and the accumulation of ECM, resulting in compromised bladder fluid storage and emptying functionality [17, 18]. Fibrotic BSMCs can transition from a contractile, nonproliferative phenotype to a synthetic phenotype, altering the interaction between BSMCs and the ECM and resulting in contractile dysfunction [19]. Remodeling of the ECM and increased expression of TGF-β1 are considered the mechanisms underlying bladder fibrosis [20]. A three-dimensional macromolecular network composed of collagen, fibronectin, and various other glycoproteins composes the bladder ECM. A complex network is created when matrix elements interact with one another and cells cling to adhesion receptors. The regular maintenance of homeostasis depends on the regulation of vital cellular processes, such as migration, growth, survival, and differentiation, by cell surface receptors, which transduce signals from the external matrix into cells. Under both normal and pathological circumstances, a number of different matrix-degrading proteins constantly remodel the ECM, which is a fluid architectural system [21]. Collagen types I and III, in fibrous form, serve as the principal scaffold matrix proteins in the ECM, contributing to structure, tensile strength, and bladder compliance in patients with fibrotic diseases [22]. A substantial increase in type III collagen may arise from enhanced synthesis, with excessive collagen deposition marking the final phase of the fibrotic process. The collagen fibers in the parietal layer of the bladder are mainly composed of collagen I, collagen III and collagen IV. Among them, the content of collagen III is the factor that determines bladder compliance. The more collagen III is deposited, the lower the bladder elasticity, leading to bladder dysfunction [23, 24]. Through the integrin cytoskeleton and other mechanisms, a highly elastic and low viscous extracellular matrix promotes the production of SMA during the progression of fibrosis [25, 26]. The results indicated that the levels of these proteins were greater in HBdSMCs treated with TGF-β1 and in rats treated with LPS than in those in the control group.

Three different TGF-β isoforms (TGF-β1, TGF-β2, and TGF-β3) have been found in animals. All three TGF-β isoforms interact with the TGFR2 receptor, which then attracts and activates the TGFR1 receptor [27]. Smad2 and Smad3 are activated by TGFβ-R1, which causes them to separate from the type I receptor and form a heterotrimeric complex with Smad4 that translocates into the nucleus. In collaboration with general transcription factors, other transcription factors, or auxiliary proteins, Smad governs the transcription of target genes [9, 28, 29]. The EMT is an intricate process governed by an extensive interactome comprising protein‒protein and gene interactions that are triggered and regulated in response to extracellular signals. At the forefront of these interactions is TGF-β1. TGF-β1 promoted SNAIL1. ZEB1 is a downstream gene of SNAIL1, and SNAIL1 also enhances ZEB1. SNAIL1 and ZEB1 inhibit E-cadherin and promote the expression of N-cadherin and vimentin [30]. These findings are consistent with our findings.

Melatonin is sleep-inducing and controls seasonal and circadian rhythms. As a multifunctional molecule, it also possesses other qualities, such as anti-inflammatory and immunomodulatory effects [31, 32]. The fibrotic response primarily encompasses four stages: initial organ injury, effector cell activation, effector cell formation, and dynamic deposition of the extracellular matrix (ECM). Melatonin modulates each of these stages and has the capacity to diminish fibrosis levels in various organs [12]. The therapeutic efficacy of melatonin against LPS-induced bladder fibrosis in rats in this study was substantiated by the mitigation of histopathological observations. The TGF-β1/Smad pathway was assessed through immunohistochemistry and western blotting. We noted elevated expression of TGF-β1, p-Smad2, and p-Smad3 in the double perfusion group compared to the sham operation group. However, melatonin alleviates these effects. Similar outcomes were noted in the *in vitro* cellular model. These data imply that the administration of melatonin leads to the downregulation of the TGF-β1/Smad pathway. We adopted the same approach to assess the degree of EMT. We noted an increase in the expression of N-cadherin and vimentin coupled with a decrease in E-cadherin in the LPS double perfusion group compared to the sham operation group. These trends improved with melatonin treatment. These results indicate that melatonin administration can alleviate EMT. Consequently, we infer that the preventive impact of melatonin on bladder fibrosis might be associated with the inhibition of the TGF-β1/Smad pathway and EMT.

We also conducted proteomic analysis utilizing HBdSMCs from the control, TGF-β1 model, and melatonin-treated groups. We identified CCN1 as a probable target protein for melatonin in the treatment of fibrosis. CCN1 is a multifunctional protein that plays a pivotal role in diverse physiological processes, including embryonic development, aging, vascular maintenance, and tissue damage repair [33, 34]. Additionally, CCN1 has been linked to a few clinical conditions, such as cancer, atherosclerosis, arthritis, and fibrosis [35–38]. CCN1 is activated by many growth factors, including TGF-β1, FGF2, and GH [39]. In contrast to normal human lung tissue, Kurundkar et al. reported a significant increase in CCN1 expression in the lung tissues of individuals with idiopathic pulmonary fibrosis. After that, they demonstrated through a number of experimental investigations that CCN1 causes lung fibrosis by boosting the expression of the TGF-1β/Smad 3 pathway [40]. Kulkarni et al. reported that CCN1 could mediate profibrotic effects by augmenting TGF-β1 signaling [41]. ZHI-QIANG LI et al. also observed a continuous increase in CCN1 expression in liver fibrosis, suggesting a potential association with the progression of liver fibrosis [42]. We have found through experimental studies that exogenous TGF-β1 activates CCN1, potentially contributing to bladder fibrosis via the TGF-β1/Smad pathway. Nevertheless, certain studies have indicated that CCN1 can exert an antifibrotic effect by fostering fibroblast senescence and apoptosis [43]. Consequently, additional research is required to elucidate the precise relationship between CCN1 and fibrosis. Furthermore, the expression of the ferroptosis gene squalene cyclooxygenase (SCOX) was significantly reduced following melatonin treatment. Consequently, SQLE may represent another target protein of melatonin in the treatment of fibrosis. SQLE is a crucial enzyme in cholesterol biosynthesis, and no pertinent reports on the role of SQLE in fibrosis have been published. Nevertheless, there are reports indicating that SQLE promotes the proliferation of colon cancer cells, fosters intestinal dysbiosis, and expedites colorectal cancer [44]. More intriguingly, studies have revealed that a decrease in squalene epoxide hydrolase can expedite the progression and metastasis of colorectal cancer [45]. In a recent study, Zhirui Zhang et al. reported significant upregulation of SQLE expression in hepatocellular carcinoma (HCC), which is associated with an adverse clinical prognosis. SQLE promotes HCC growth, epithelial–mesenchymal transition, and metastasis by activating TGF-β/SMAD signaling [46]. We found by immunoblotting that SQLE expression was substantially increased in the TGF-β1 group and the LPS group, whereas its expression was significantly decreased following MEL treatment. Hence, SQLE represents another pivotal protein that will be a focal point in our future investigations.

Studies have shown that the melatonin receptors MT1 and MT2 are expressed mainly in the mast cell network of the lamina propria and muscle layer of the bladder. [47, 48]Many studies have shown that melatonin receptors are expressed on inflammatory cells, such as macrophages and T lymphocytes, and are also expressed on white blood cells. In fact, melatonin has two different effects on regulating inflammation. Melatonin can both promote the occurrence of inflammation and inhibit the inflammatory response. This process depends on many factors, including the different cells involved, the microenvironment, and the severity of inflammation. The ability of melatonin to slow fibrosis may be attributed to the inhibition of oxidative stress and inflammatory signals. These processes include downregulation of iNOS and COX-2 expression, inhibition of NF-κB and inflammasome NLRP3 activation, and upregulation of Nrf2 [32, 49–54]. In the present study, we observed obvious inflammatory cells in fibrotic rat bladder tissue, and the degree of inflammation was significantly reduced after melatonin treatment. However, the possibility that melatonin may directly act on receptors cannot be ignored, although this possibility has not been confirmed by research.

Our study yielded the following primary findings: first, we established a rat model of chronic inflammation-induced bladder fibrosis through continuous intravesical instillation of PS and LPS over a 6-week period; second, LPS activation induces bladder injury via the TGF-β1/Smad and EMT pathways, which play pivotal roles in the pathogenesis of bladder fibrosis. Third, melatonin modulation of the associated TGF-β1/Smad pathway and EMT effectively alleviated chronic inflammation-induced bladder fibrosis in rats. This study is subject to certain limitations. First, the outcomes of this study were centered on fibrosis induced by chronic inflammation. The therapeutic efficacy of melatonin was not assessed in alternative bladder fibrosis models, including neurogenic and outlet obstruction models. Second, given the intricate pathogenesis of patients with bladder fibrosis, these investigations have been limited to *in vitro* and animal studies. There is a considerable distance to cover before these treatments translate into clinical health benefits, and the translational relevance of experimental findings to humans remains constrained. Finally, subsequent functional verification and mechanistic investigation of CCN1 have yet to be performed, and relevant efforts are currently underway in our laboratory.

The pathophysiological mechanism underlying bladder fibrosis has not been fully elucidated, and the therapeutic outcomes are suboptimal. The pathophysiological mechanisms of bladder fibrosis are not fully understood, and current treatments are ineffective. In the present study, melatonin-based treatment mitigated fibrosis in a rat model of LPS-induced fibrosis. Given the limited therapeutic options for chronic inflammation and fibrosis of the bladder, melatonin might represent a novel therapeutic approach, with CCN1 potentially serving as a key therapeutic target.

## 5. Conclusions

Melatonin is a promising new treatment for chronic cystitis and bladder fibrosis, and CCN1 serves as a key therapeutic target.

## Supporting information

S1 Table Protein identification list. (XLSX)

S1 Fig Pearson correlation coefficient analysis and representative western blot showing SQLE expression. (A) Heatmaps were generated by calculating the Pearson correlation coefficient between all sample pairs. (B) Western blot analysis of changes in SQLE protein expression in HBdSMCs and rat bladder tissues. The data are shown as the mean ± SEM. #P<0.05, ##P<0.01, ###P<0.001 vs. the control group. *P<0.05, **P<0.01, **P<0.001 vs. the model group.
(TIF)

S1 Raw images The original western blotting image data. (PDF)

## Abbreviations

BOO: bladder outlet obstruction
BSMCs: bladder smooth muscle cells
CCN1: cellular communication network Factor 1
COX 2: cyclooxygenase-2
DMEM: Dulbecco’s modified eagle medium
DMSO: dimethyl sulfoxide
ECM: extracellular matrix
EMT: epithelial–mesenchymal transition
EMT-TF: epithelial–mesenchymal transition-regulating transcription factor
FBS: forward-based system
HBdSMCs: human bladder smooth muscle cells
LPS: lipopolysaccharide
MEL: melatonin
MT: melatonin receptor
NF-κB: nuclear factor kappa-B
NLRP3: NOD-, LRR- and pyrin domain-containing protein 3
NOS: nitric oxide synthase
NRF2: nuclear factor-E2-related factor-2
PBS: phosphate-buffered saline
PS: protamine sulfate
SD: Sprague‒Dawley
SQLE: squalene epoxidase
TGF: transforming growth factor
TGF-β: transforming growth factor-β
