# Supplementary figures and images for: Melatonin serves as a novel treatment in bladder fibrosis through TGF-β1/Smad and EMT
Source: PLoS One. 2024 Mar 13;19(3):e0295104. doi: 10.1371/journal.pone.0295104 (PMC10936792; doi:10.1371/journal.pone.0295104)

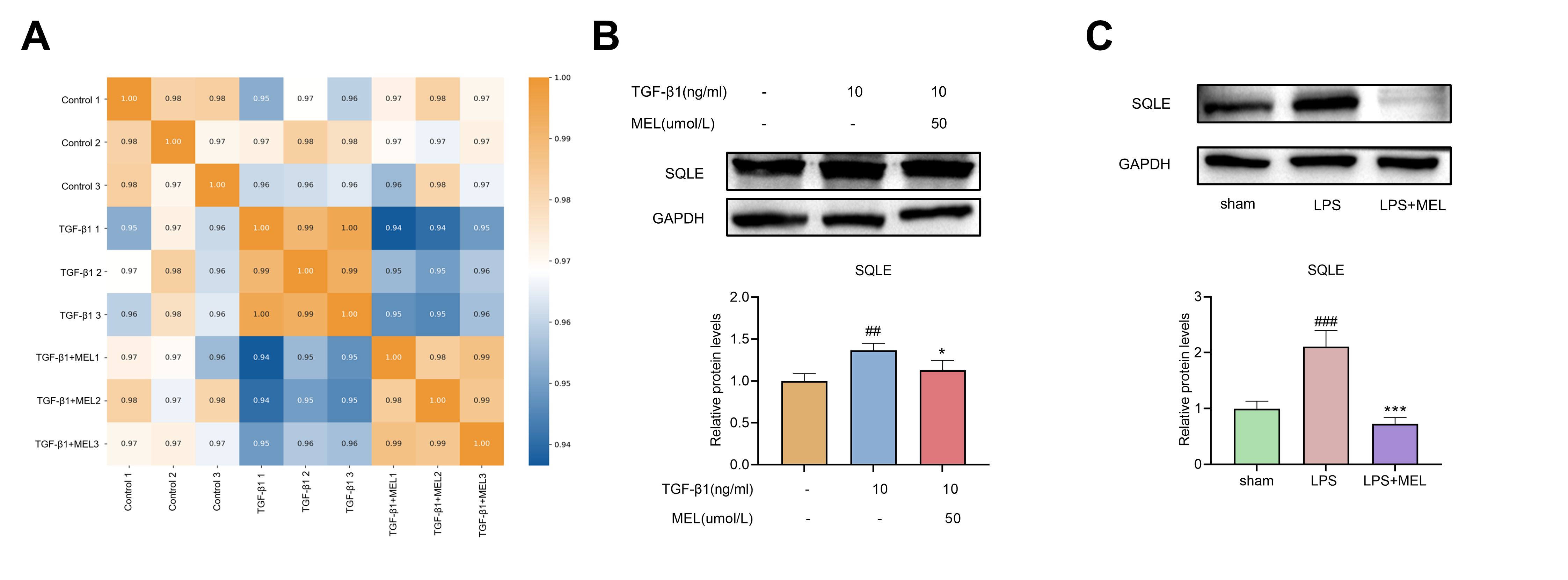

Supplement: S1 Fig — (A) Heatmaps were generated by calculating the Pearson correlation coefficient between all sample pairs. (B) Western blot analysis of changes in SQLE protein expression in HBdSMCs and rat bladder tissues. The data are shown as the mean ± SEM. #P<0.05, ##P<0.01, ###P<0.001 vs. the control group. *P<0.05, **P<0.01, **P<0.001 vs. the model group. (TIF) [file pone.0295104.s002.tif]

Fig 2C

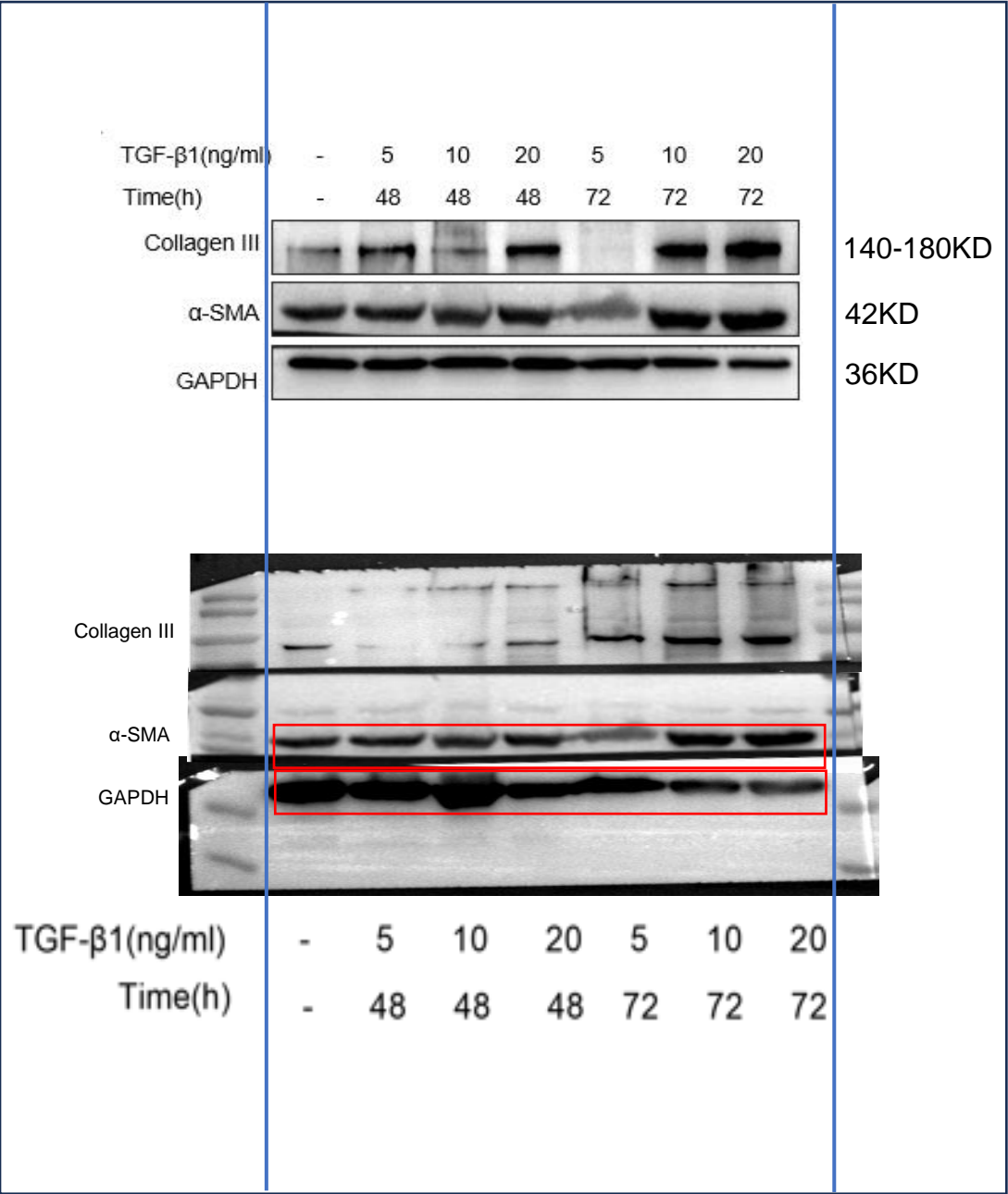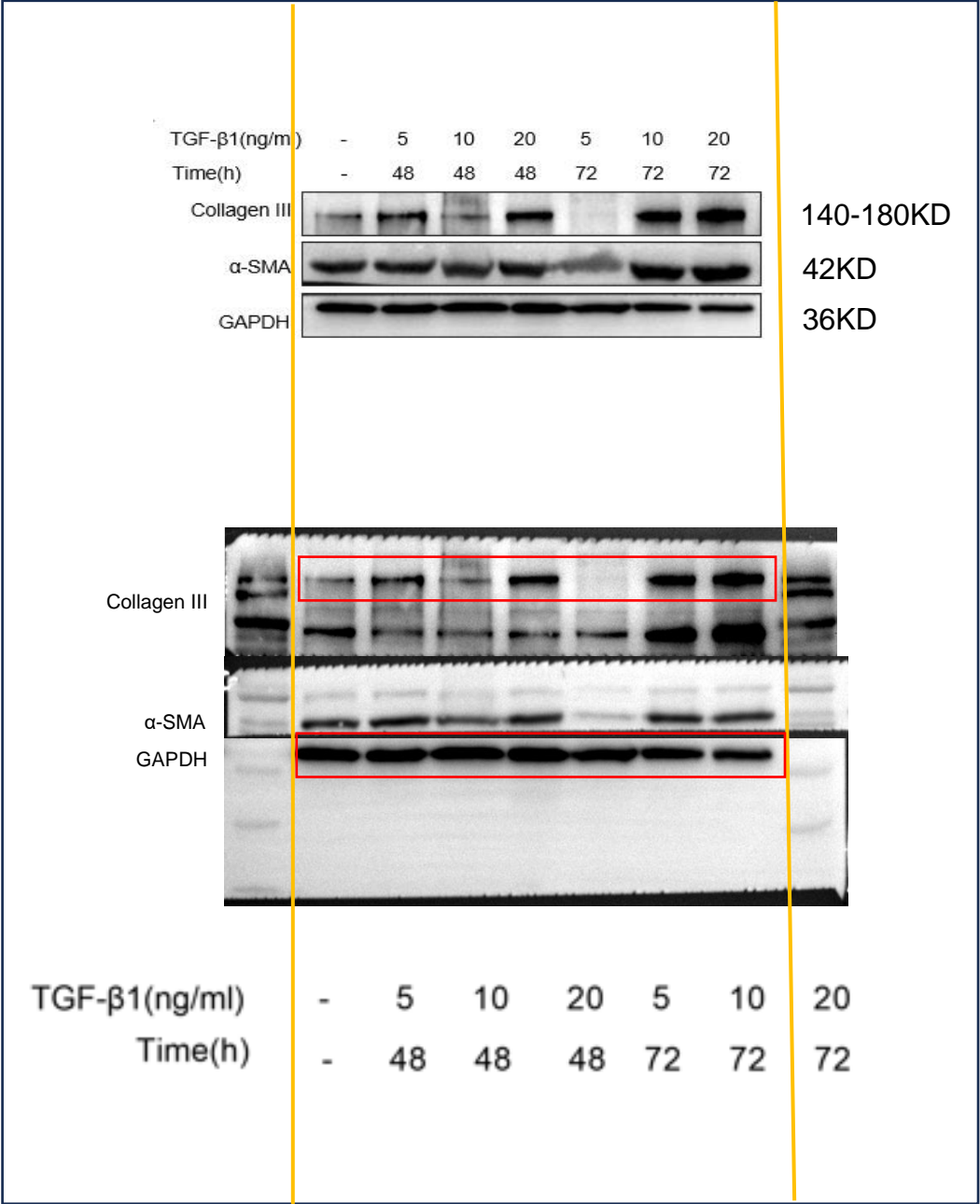

Fig 2F

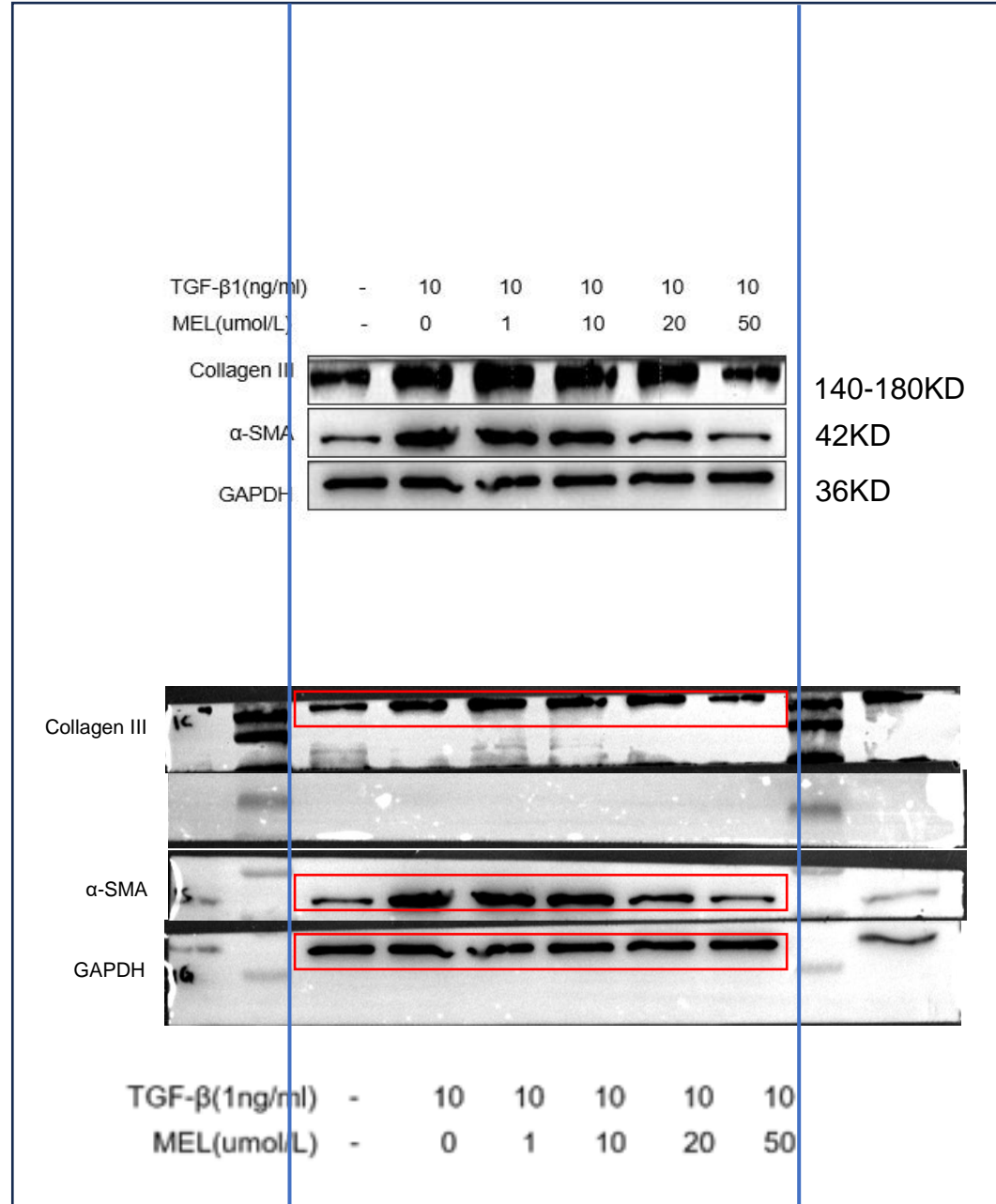

Fig 3A

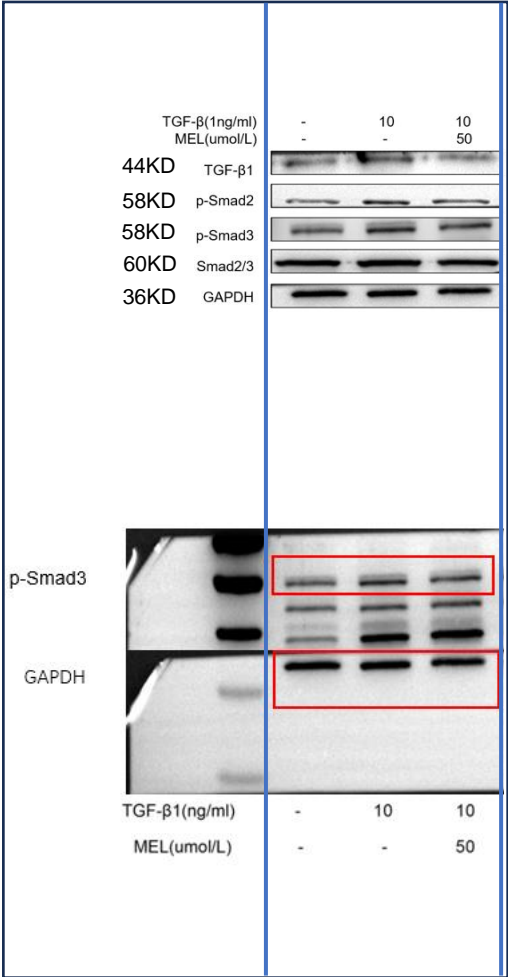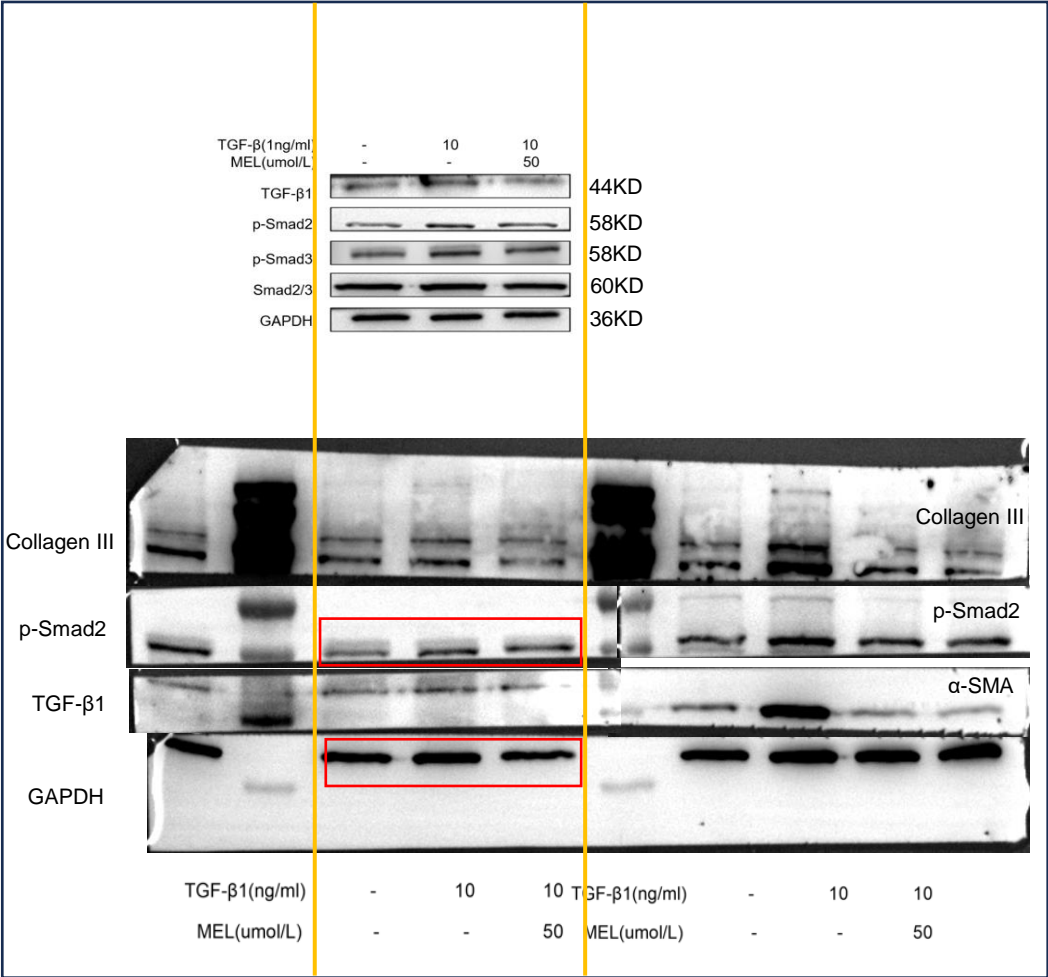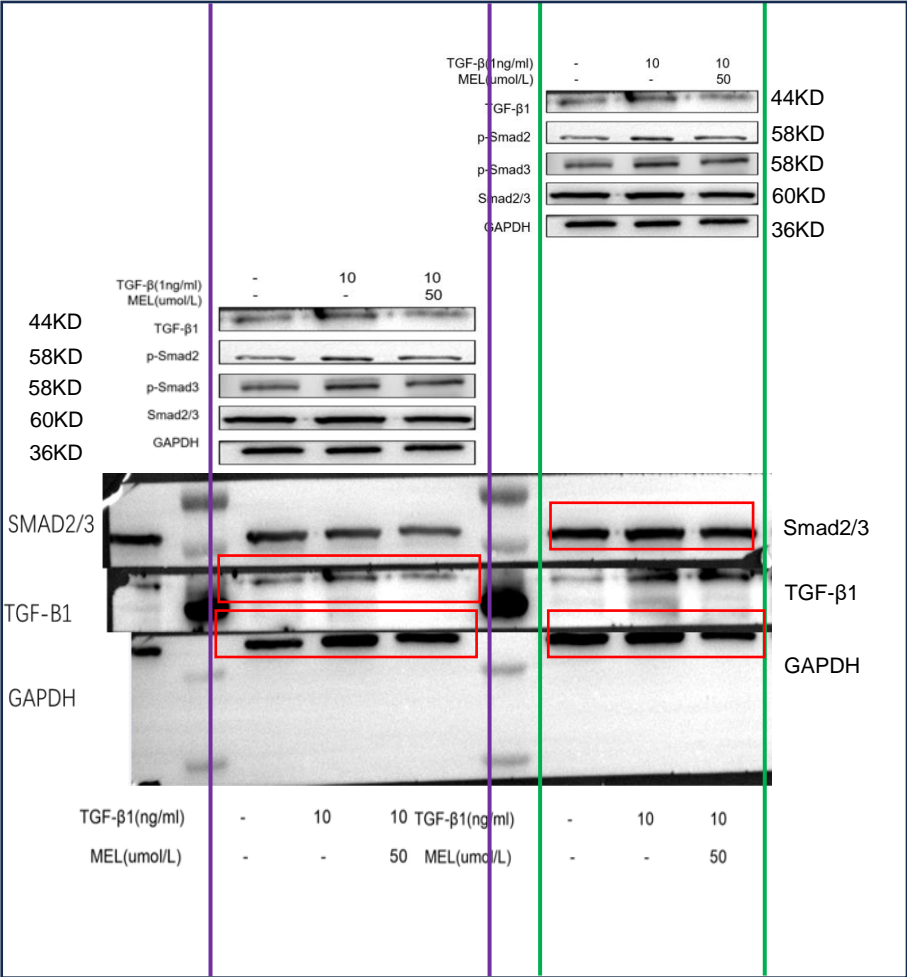

Fig 3B

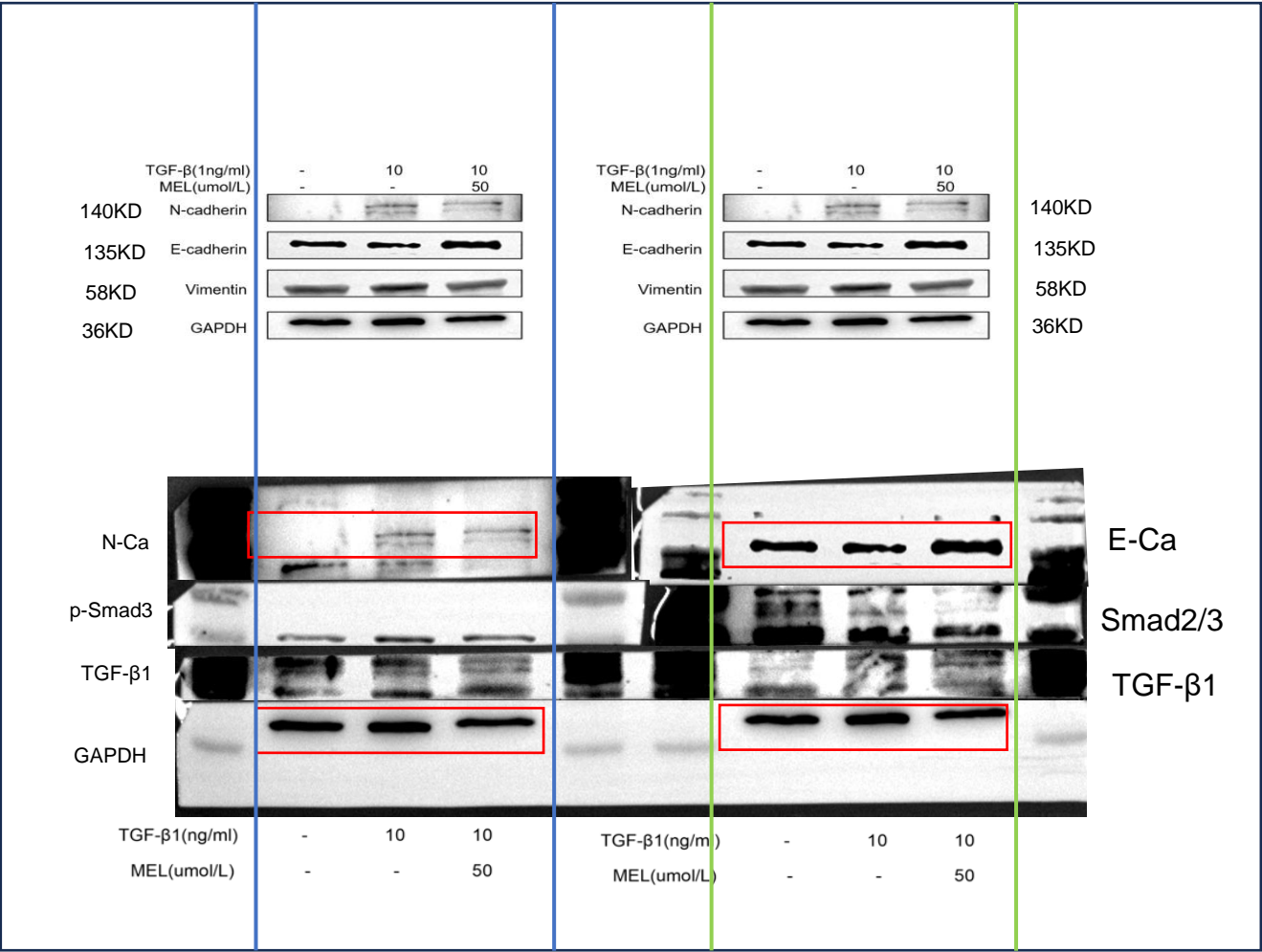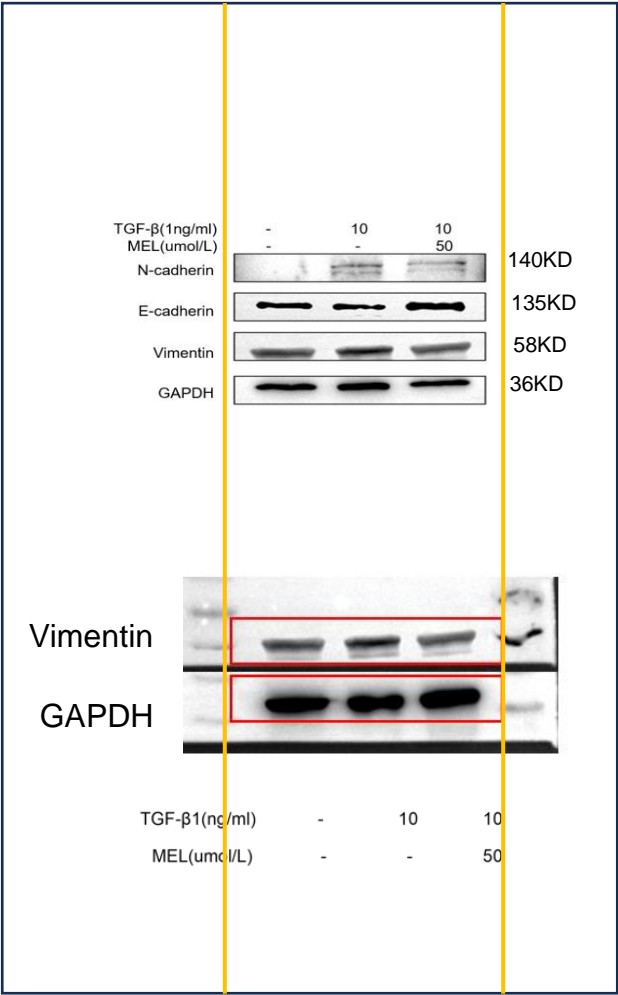

Fig 4

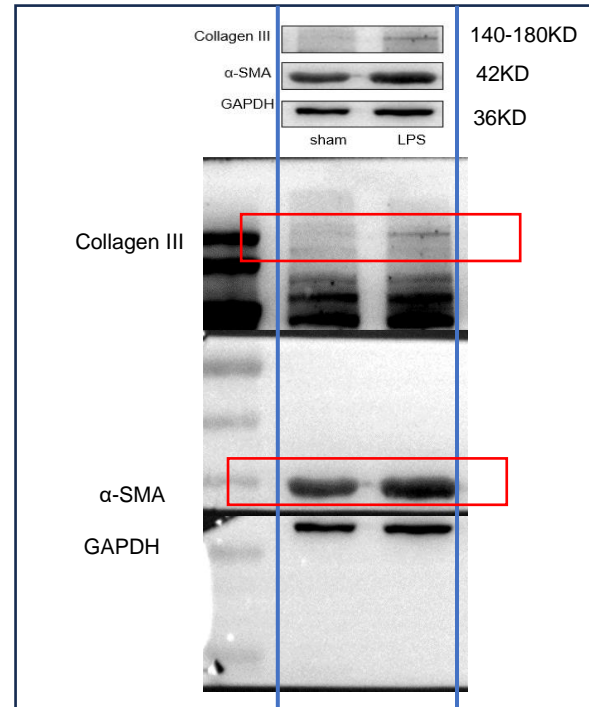

Fig 5

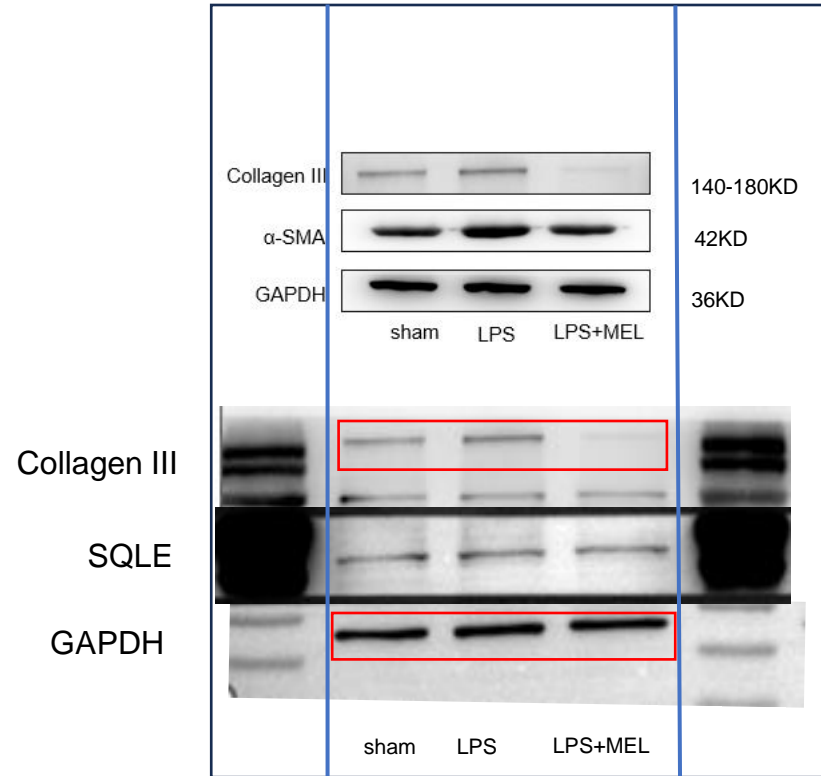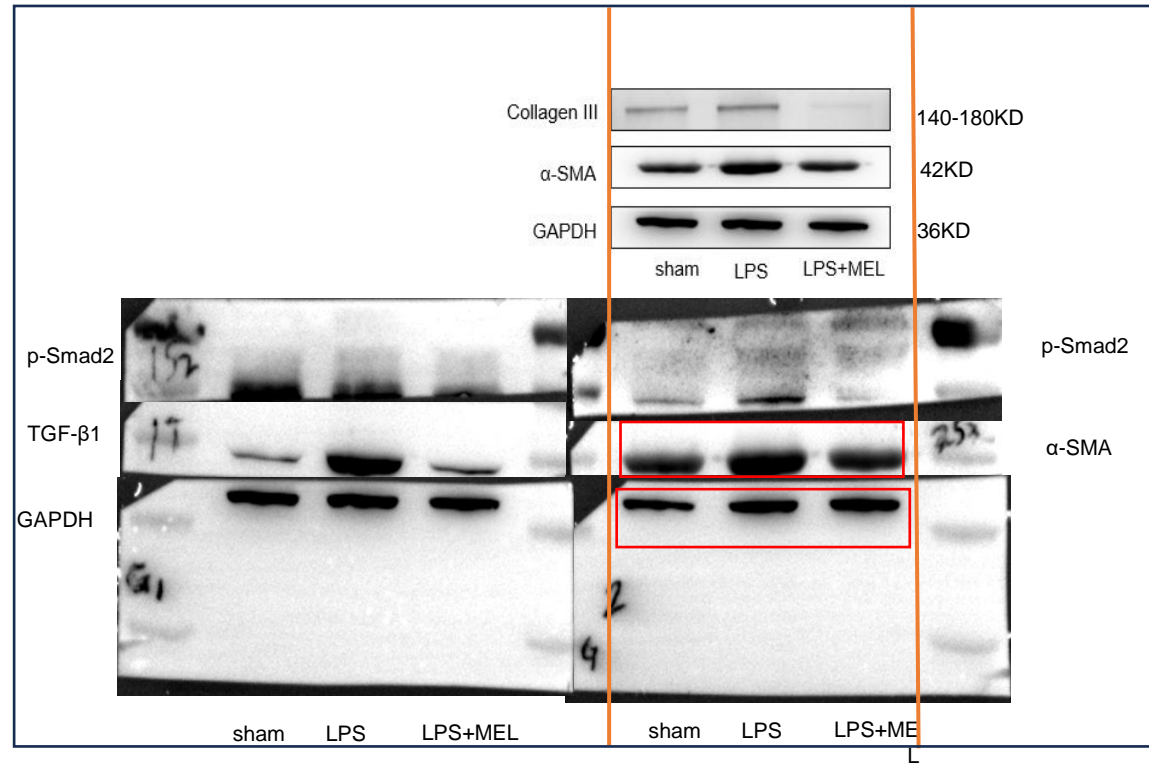

Fig 6A

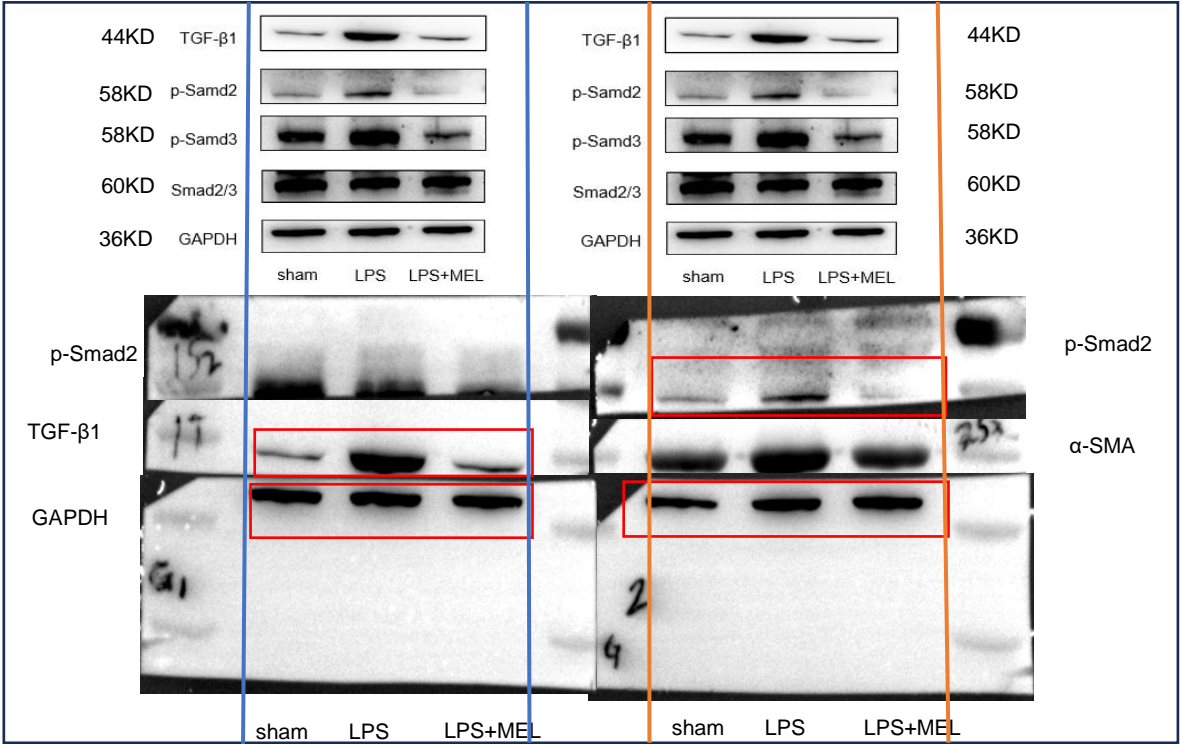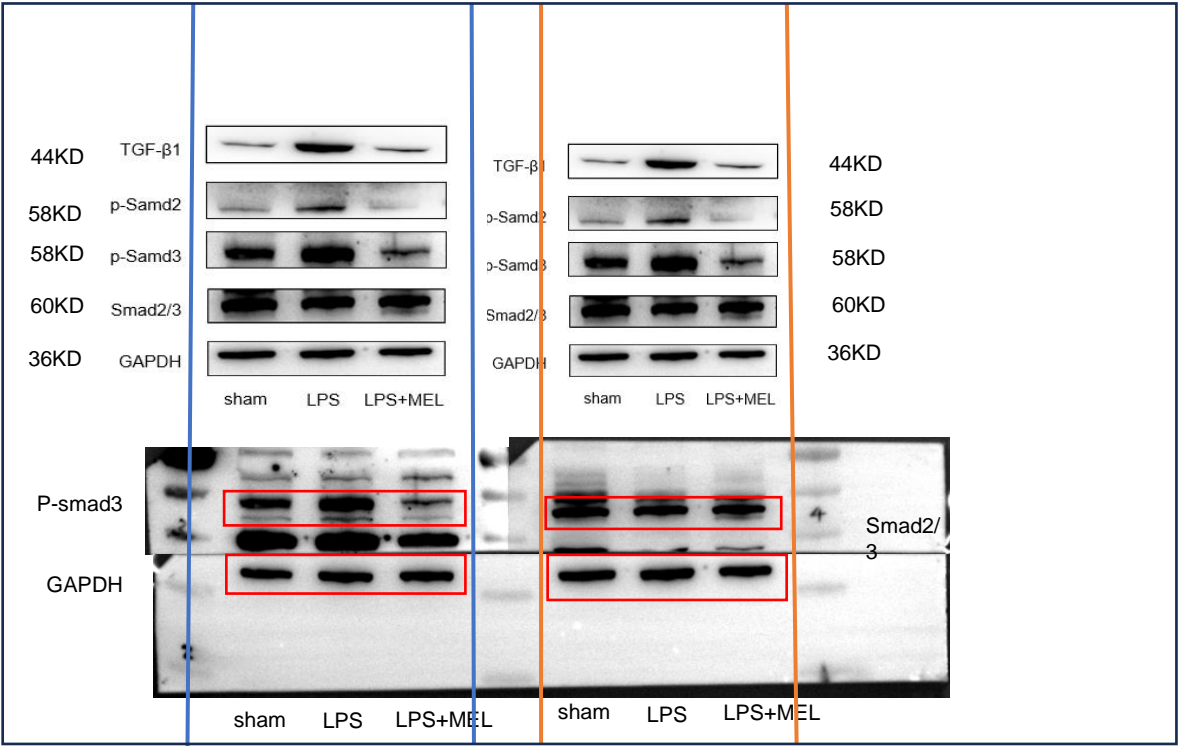

Fig 6B

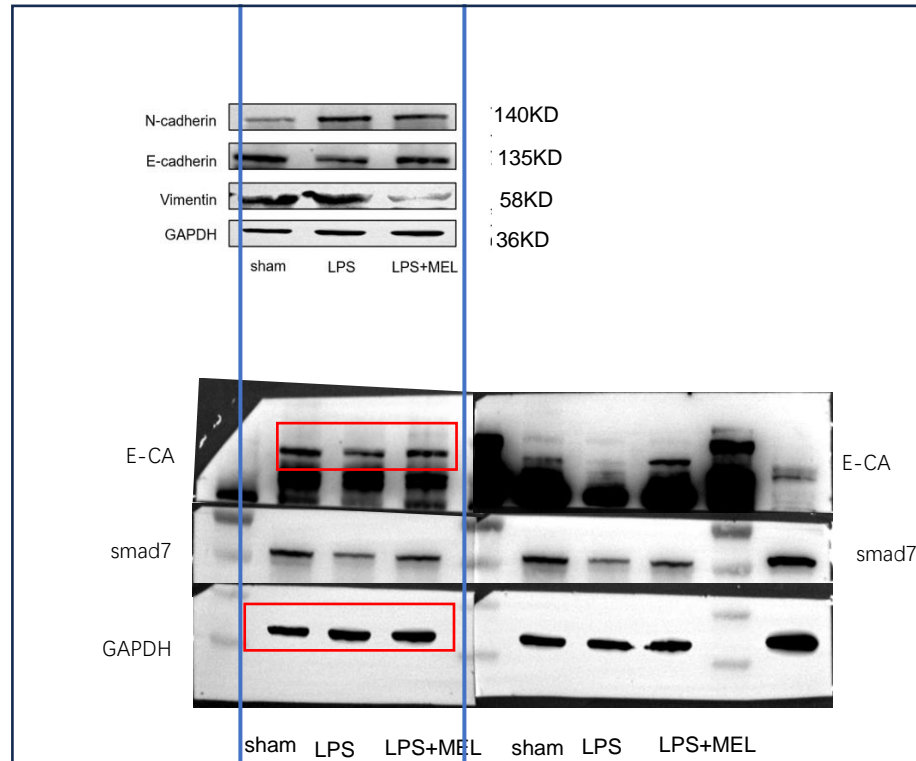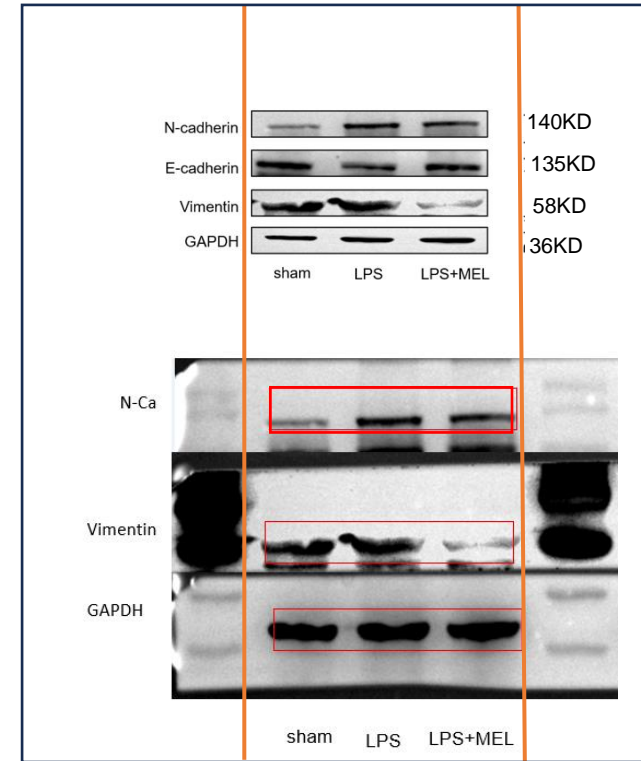

Fig 7

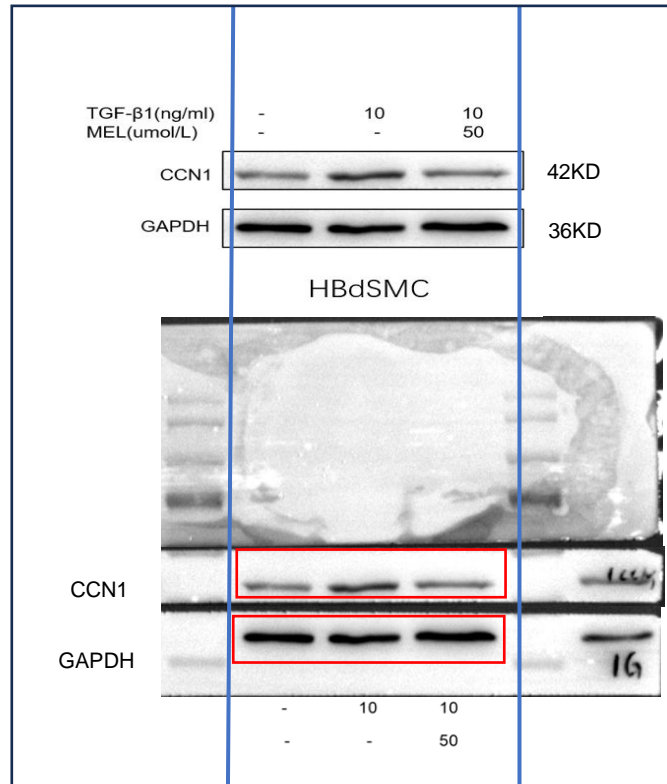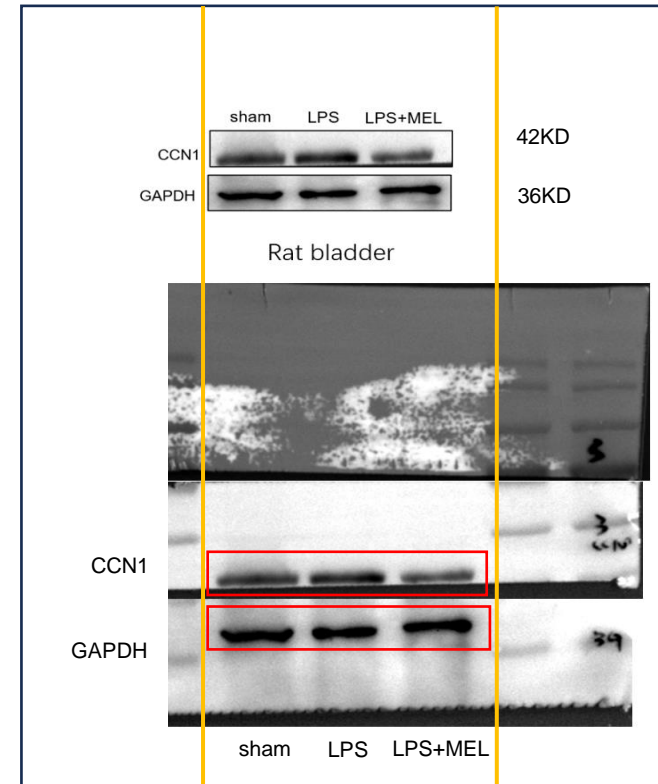

Fig S1B

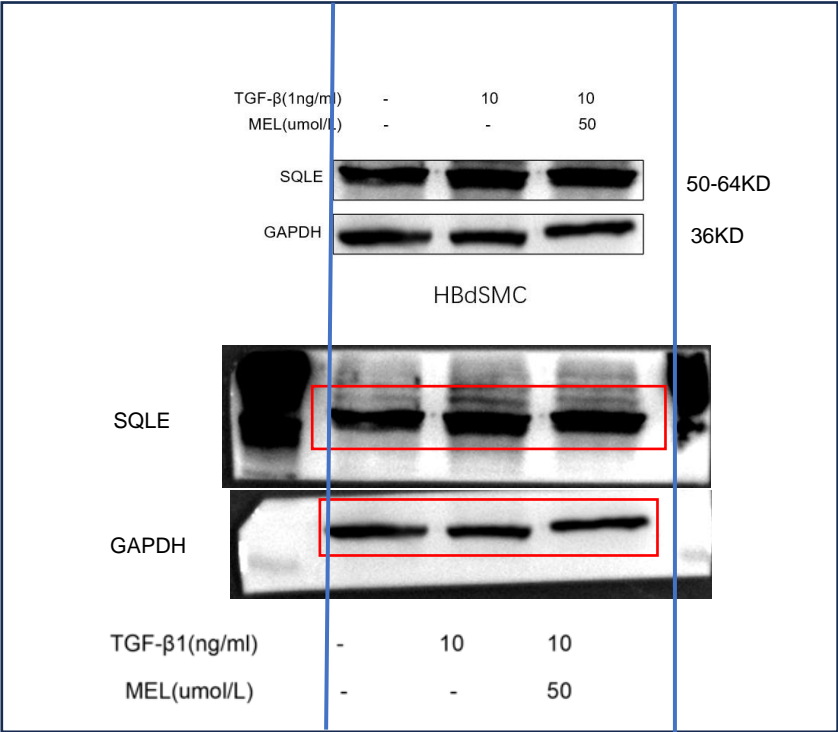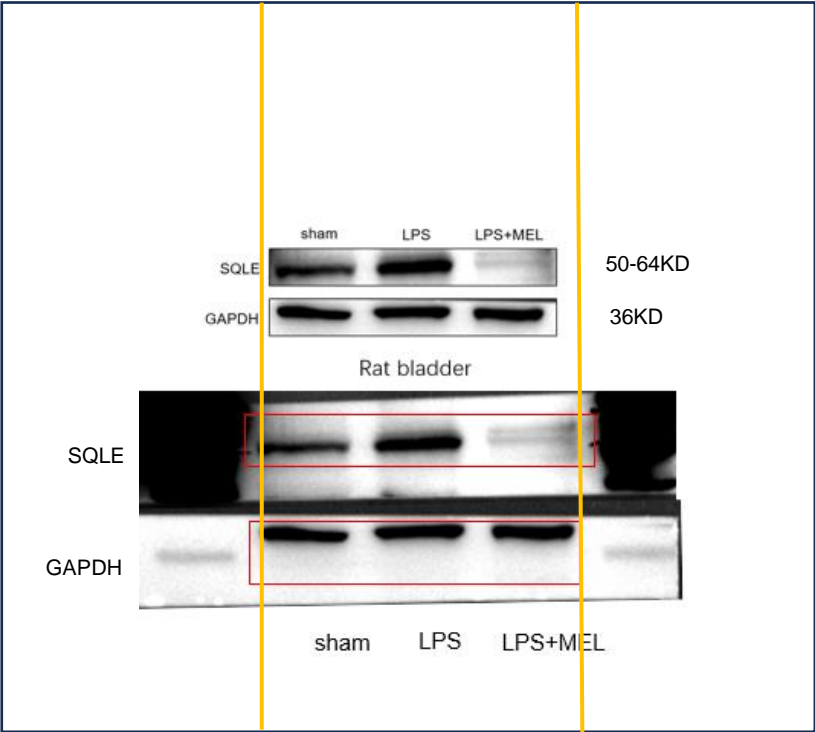

Supplement: S1 Raw images — (PDF) [file pone.0295104.s003.pdf]
